# Mixed yeast communities contribute to regionally distinct wine attributes

**DOI:** 10.1093/femsyr/foad005

**Published:** 2023-02-01

**Authors:** Diana Lynne Hawkins, Jess Ryder, Soon A Lee, Katie Parish‐Virtue, Bruno Fedrizzi, Matthew R Goddard, Sarah J Knight

**Affiliations:** School of Biological Sciences, University of Auckland, Private Bag 92019, Auckland Mail Centre, Auckland 1142, New Zealand; School of Biological Sciences, University of Auckland, Private Bag 92019, Auckland Mail Centre, Auckland 1142, New Zealand; School of Biological Sciences, University of Auckland, Private Bag 92019, Auckland Mail Centre, Auckland 1142, New Zealand; School of Chemical Sciences, University of Auckland, Private Bag 92019, Auckland Mail Centre, Auckland 1142, New Zealand; School of Chemical Sciences, University of Auckland, Private Bag 92019, Auckland Mail Centre, Auckland 1142, New Zealand; School of Biological Sciences, University of Auckland, Private Bag 92019, Auckland Mail Centre, Auckland 1142, New Zealand; School of Life and Environmental Sciences, University of Lincoln, Brayford Pool, Lincoln LN6 7DL, United Kingdom; School of Biological Sciences, University of Auckland, Private Bag 92019, Auckland Mail Centre, Auckland 1142, New Zealand

**Keywords:** *terroir*, wine, yeast, fermentation, Pinot Noir, New Zealand

## Abstract

There is evidence that vineyard yeast communities are regionally differentiated, but the extent to which this contributes to wine regional distinctiveness is not yet clear. This study represents the first experimental test of the hypothesis that mixed yeast communities—comprising multiple, region-specific, isolates, and species—contribute to regional wine attributes. Yeast isolates were sourced from uninoculated Pinot Noir fermentations from 17 vineyards across Martinborough, Marlborough, and Central Otago in New Zealand. New methodologies for preparing representative, mixed species inoculum from these significantly differentiated regional yeast communities in a controlled, replicable manner were developed and used to inoculate Pinot Noir ferments. A total of 28 yeast-derived aroma compounds were measured in the resulting wines via headspace solid-phase microextraction coupled with gas chromatography-mass spectrometry. Yeast community region of origin had a significant impact on wine aroma, explaining ∼10% of the observed variation, which is in line with previous reports of the effects of region-specific *Saccharomyces cerevisiae* isolates on Sauvignon Blanc ferments. This study shows that regionally distinct, mixed yeast communities can modulate wine aroma compounds in a regionally distinct manner and are in line with the hypothesis that there is a microbial component to regional distinctiveness, or *terroir*, for New Zealand Pinot Noir.

## Teaser

This study shows that mixed yeast communities of vineyards impact wine aroma in a regionally distinct manner in New Zealand Pinot Noir.

## Introduction

Wine is well known for its regional distinctiveness, with the same grape varieties grown in different localities exhibiting different attributes. Regional distinctiveness is a point of differentiation for certain consumers, and thus distinctiveness can have economic value for wine producers (Van Leeuwen and Seguin 2006). Historically, regional distinctiveness, or *terroir*, has been attributed to regional differences in climate, soil, annual weather patterns, aspect, and cultural vineyard practices, among other factors, but a role for microbes has not been considered (Van Leeuwen and Seguin 2006, Alexandre 2020). However, an increasing number of studies across a number of countries have demonstrated that viticultural regions harbour regionally distinct microbial communities (Gayevskiy and Goddard 2012, Bokulich et al. 2013, 2016, Knight et al. 2015, Griggs et al. 2021). This, combined with evidence that different species and strains of yeast impart distinct flavours and aromas to wine (Howell et al. 2004, Swiegers and Pretorius 2005, Sumby et al. 2010, Hall et al. 2011, 2017, Tempère et al. 2018) suggests that microbes may contribute to a wine’s regional distinctiveness, or *terroir*.

Key wine aroma compounds, such as esters, higher alcohols, carbonyl compounds, sulfur compounds, volatile phenols, and volatile acids, have been directly linked to yeasts’ metabolic processes during fermentation (Howell et al. 2004, Swiegers and Pretorius 2005, Sumby et al. 2010, Zott et al. 2011, Franc et al. 2017, Tempère et al. 2018, Kinzurik et al. 2020). The production of these compounds has been found to vary amongst yeast species and strains, resulting in differences in the type and quantity of aroma compounds in wines fermented by different species and strains (Howell et al. 2004, Swiegers and Pretorius 2005, Sumby et al. 2010, Hall et al. 2011, 2017, Tempère et al. 2018). Further, in ferments with more than one species or strain of yeast, interactions between yeasts, including metabolite sharing, may further modulate final wine aroma (Bordet et al. 2020) and this variance in aroma cannot be replicated by simply blending together the wines produced by individual species or strains (Howell et al. 2006, Anfang et al. 2009). If specific combinations of different yeasts produce specific types and amounts of metabolites, and there is evidence for different specific combinations of unique yeasts (communities) in different regions, it is reasonable to predict that this can result in a microbial aspect to *terroir*.

Many studies report that vineyard yeast are transported to wineries on grapes, are present in grape must, and contribute to wine fermentations (Fleet 2003, Grainger and Tattersall 2016, Martiniuk et al. 2016, Hall et al. 2017, Morrison-Whittle and Goddard 2018). Consequently, uninoculated fermentations are a way of capturing the contributions of local yeast communities during fermentation (Sumby et al. 2010, Gayevskiy and Goddard 2012, Bokulich et al. 2013, Medina et al. 2013, Taylor et al. 2014, Šuranská et al. 2016). Such uninoculated fermentations typically comprise multiple yeast species, whose population numbers, species type, and strains are often hard to characterize (Povhe Jemec et al. 2001, Selli et al. 2005, Goddard 2008, Zott et al. 2008, Medina et al. 2013, Šuranská et al. 2016, Bagheri et al. 2017). Multiple yeast species of varying ethanol tolerances are present at the beginning of uninoculated fermentations (Povhe Jemec et al. 2001, Selli et al. 2005, Di Maro et al. 2007, Goddard 2008, Zott et al. 2008, Bokulich et al. 2013, Medina et al. 2013, Šuranská et al. 2016, Bagheri et al. 2017, Stefanini and Cavalieri 2018). As fermentations progress, in addition to metabolites that modulate wine aroma, some yeast species produce toxins and ethanol allowing them to outcompete others (De Deken 1966, Young and Yagiu 1978, Povhe Jemec et al. 2001, Goddard 2008, Ciani and Comitini 2015, Šuranská et al. 2016, Tempère et al. 2018). If present, *Saccharomyces* species, particularly *Saccharomyces cerevisiae*, are responsible for the fermentation of most sugars due to their ability to produce and tolerate increasing ethanol and elevated temperatures (Swiegers and Pretorius 2005, Thomson et al. 2005, Di Maro et al. 2007, Goddard 2008, Šuranská et al. 2016, Varela and Borneman 2017, Englezos et al. 2018). Consequently, the diversity of species is typically greater at the early stages of fermentation (Selli et al. 2005, Goddard 2008, Zott et al. 2008).

There are some compelling studies that have shown correlations between regional differences in grape microbiomes and wine metabolomes (e.g. Bokulich et al. 2016, Drumonde-Neves et al. 2017), but correlation does not demonstrate causation as another region-specific factor may have driven differences in both the microbiomes and wine chemistry. However, objective, controlled direct experiments to test whether there is a microbial aspect to *terroir* are limited (Alexandre 2020). Empirical tests of whether the entire grape-associated microbiome contributes to regional wine attributes would be impossible as most grape microbes do not grow on artificial laboratory media. One estimate is that 95% of wine grape-associated fungi do not grow on standard media (Taylor et al. 2014), and therefore, it is not currently possible to isolate and grow the total microbial community from the fruits or juice to derive an experimental inoculum to conduct such tests. However, most yeast components of the grape microbiome are able to be cultured in the laboratory. Yeast communities that derive from the local environment are abundant in spontaneous ferments (Taylor et al. 2014, Morrison-Whittle and Goddard 2018), but also contribute to inoculated ferments that are sulfured, and thus locally derived yeast communities variously contribute to fermentation (Povhe Jemec et al. 2001, Selli et al. 2005, Goddard 2008, Zott et al. 2008, Medina et al. 2013, Šuranská et al. 2016, Bagheri et al. 2017).

Sauvignon Blanc fermented by regionally distinct populations of *S. cerevisiae*, the work horse of wine fermentation, provided the first and only experimental evidence of microbially driven regional distinctions in wine phenotypes that we are aware of (Knight and Goddard 2015, Knight et al. 2015); however, as discussed, wine fermentation is more complex (Selli et al. 2005, Goddard 2008, Zott et al. 2008) and we are aware of no tests as to whether regionally distinct yeast communities produce different wine chemistries or not.

The Martinborough, Marlborough, and Central Otago regions represent 85% of New Zealand’s Pinot Noir production (New Zealand Wine Growers 2022a) and are known to vary by climate, soil, geography, and crucially vineyard-associated yeast communities (Knight and Goddard 2015, Morrison-Whittle and Goddard 2015). Thus, NZ Pinot Noir provides an excellent system to test and quantify whether mixed yeast communities contribute to wine regional distinctiveness. To evaluate the impact that mixed yeast communities have on wine aroma, yeasts were isolated from each of the regions and representative communities were reconstructed and then inoculated into a standardized Pinot Noir grape juice. Here, we test the hypothesis that mixed yeast communities—comprising multiple, region-specific, culturable isolates, and species—contribute to regional wine attributes. While simplified from the true complexity of the fruit and ferment microbial environment, using representative culturable yeast communities enables these naturally occurring ecosystems to be emulated in a controlled manner (De Roy et al. 2014, Ponomarova and Patil 2015). This not only allows objective empirical tests of this hypothesis but also provide potential practical tools for winemakers. As far as we are aware, this is the first time mixed yeast communities have been objectively tested for their contribution to *terroir*.

## Methodology

### Regional yeast community isolation

**Figure 1.**
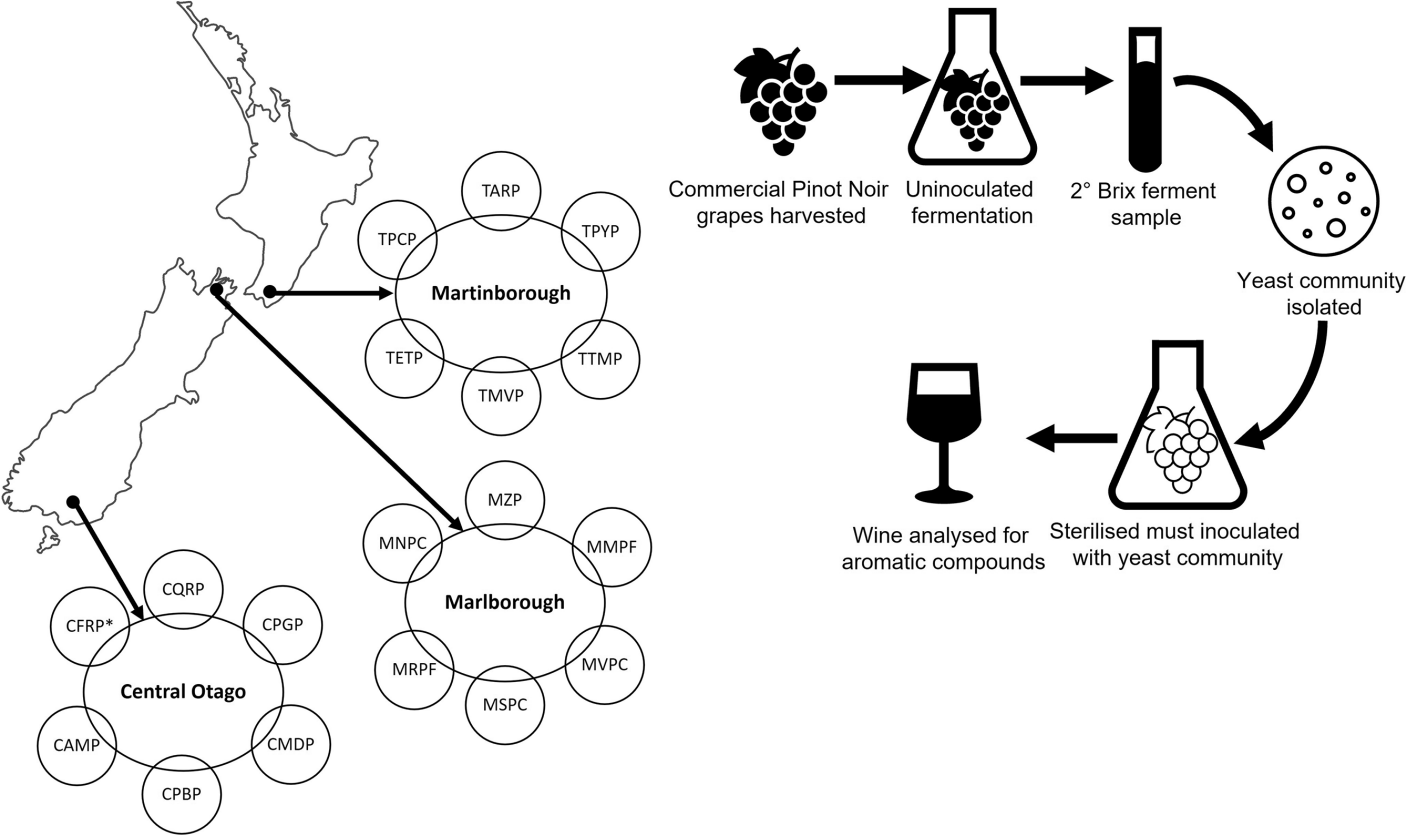
Location of the New Zealand Pinot Noir growing regions tested in this study and the site codes of the respective vineyard sites, where fruit was collected from to isolate the regional yeast communities with experimental design. *While all 18 vineyard sites are shown here, the yeast community was unable to be isolated from site CFRP in Central Otago, resulting in 17 yeast communities for the subsequent fermentation trial, as described in the methods.

Fruit was collected from six Pinot Noir vineyards in each of the three geographic regions tested ∼2 days before commercial harvest (Fig. 1). Within each vineyard site, grapes were collected and pooled from nine focal vines that captured the topological variability observed. Fruit was collected into sterile plastic bags using snips sterilized with Trigene (10% v/v), chilled to 4°C, and transported to the University of Auckland for processing.

In the laboratory, a total of 20 kg of fruit from each vineyard site was weighed, hand destemmed using sterile gloves, and combined into sterile 20 l fermentation vessels for each vineyard. The grapes were crushed by hand within the vessel and the starting Brix and yeast assimilable nitrogen (YAN) were measured. The YAN was adjusted to a minimum of 200 mg/l using diammonium phosphate; if the YAN was above 200 mg/l no diammonium phosphate addition was made. The ferments were warmed to 20 ± 3°C to initiate uninoculated fermentation and the ferment weights (El Haloui et al. 1988).

Brix and temperature were monitored daily to track fermentation progress. A 10 ml sample to capture the yeast communities present early in these ferments were taken after a reduction of 2° Brix. The composition of the yeast community changes dramatically during fermentation and reduces in diversity (Goddard et al. 2010). By sampling as the ferment begins to accelerate, we aimed to capture the widest diversity of isolates that actively metabolize during fermentation, while excluding those that were incidentally present on the grapes but do not contribute to fermentation. Unfortunately, the desired sampling time point for vineyard CFRP in Central Otago was missed, which reduced the number of regional yeast communities from this region to five, rather than six. Samples were stored in 15% (v/v) glycerol at −80°C to preserve the yeast communities prior to isolation.

Frozen must samples were thawed, and serial dilutions were plated onto YPD (1% yeast extract, 2% peptone, 2% glucose, and 2% agar) and incubated at 28°C for 48 hours. Single colonies were selected in an unbiased manner using a grid-like process until 96 individual isolates were obtained from each sample, or all viable single colonies were selected, whichever occurred first. This resulted in a mixed yeast community of up to 96 individual yeast isolates from each vineyard site. Individual isolates were stored in 96-well culture plates in 15% glycerol at −80°C until further analysis.

### Taxonomic identification of representative yeast communities

Frozen yeast isolates were revived in liquid YPD and DNA was extracted using 1.25 mg/ml Zymolyase in 1.2 M sorbitol and 0.1 M KH_2_PO_4_ at pH 7.2 at 37°C for 30 minutes followed by 10 minutes at 95°C and 1 minute at 15°C to lyse the cells (Knight and Goddard 2015). The ITS1-5.8S rRNA-ITS2 region was amplified via PCR using the ITS1 and ITS4 primers (White et al. 1990) following (Goddard 2008). Following amplification, HaeIII and Hinf1 restriction enzymes were used separately to digest the ITS amplicons (Esteve-Zarzoso et al. 1999). The digested ITS fragments were visualized by gel electrophoresis and isolates were grouped into cohorts based on visual assessment of band patterns (Esteve-Zarzoso et al. 1999).

Three individuals from each cohort were selected for Sanger sequencing such that isolates from each geographic region were equally represented. If individuals within a cohort were not found across all three regions, three individuals were taken from three different vineyard sites to avoid selecting clones. PCR amplification of the D1/D2 26S rRNA region was performed following Gayevskiy and Goddard (2012), amplicons were cleaned via NucleoSpin Gel and a PCR clean-up kit (Macherey-Nagel) and sequenced via dye terminator Sanger sequencing at Auckland Genomics at the University of Auckland. Once sequenced, the DNA fragments were subjected to BLAST analyses against the NCBI nucleotide database to identify the species of the microorganism in question.

### Lab-scale fermentation to test contribution of regional yeast communities to *terroir*

#### Grape must preparation and sterilization

Pinot Noir juice and solids for the fermentation trials were prepared from frozen commercially produced Pinot Noir grapes from the 2018 harvest from across the Martinborough, Marlborough, and Central Otago regions. Fruit was thawed, hand-destemmed, macerated, and mixed to create a standardized homogenized must. A 20% v/v solution of dimethyl dicarbonate (DMDC) in ethanol was applied at a rate of 300 μl/l to sterilize the grape must (Daudt and Ough 1980, Delfini et al. 2002, Costa et al. 2008) for 8 hours at 22 ± 1°C. Aliquots of 100 ml of juice were transferred along with 25 ml of grape skins and seeds into sterilized tubes and stored at −80°C until required.

The day before inoculation, the frozen must aliquots were thawed and an additional DMDC treatment was employed at a rate of 200 μl/l overnight at 22 ± 1°C. The following morning the must was placed in a cold room. A total of 2 hours before inoculation the must was put at room temperature to warm.

#### Yeast community preparation

Isolates were revived from frozen 96-well culture plates by transferring via a flame sterilized, 96-well pin microplate replicator to another 96-well culture plate containing liquid YPD, which was then incubated at 28°C for 72 hours. This extended period for time allowed each isolate to grow in isolation to maximum cell density. Immediately prior to inoculation, all 96 isolates representing a vineyard community were mixed. Since the yeast were isolated in a random manor from the original ferment sample, the 96 yeast represent not only the species diversity of the most abundant yeast at the time of sampling, but also the proportion in which they existed in the original ferment community. By growing the communities in isolation to maximum cell density first, then mixing immediately prior to inoculation, the original species composition and relative abundances of each isolate can be replicated for these mixed yeast community inoculations. The yeast mixed for each vineyard was then centrifuged at 3000 *g* for 5 minutes to pellet the cells, which were resuspended in 5 ml of sterile water ready for inoculation.

#### Inoculation and fermentation

Zip® 350 ml coffee plungers (French presses) were autoclaved and used as fermentation vessels to mimic commercial red wine production methods (Sparrow and Smart 2015). Prepared must was thawed and added to each plunger to provide a total ferment volume of 250 ml. Specific gravity (Brix) and temperature were recorded. A 100 μl sample was taken from the negative control, serially diluted, and plated on YPD agar to quantify any ambient yeast present in the must after all sterilization steps had been completed. Triplicate OD measurements of the yeast community inoculums from each vineyard site were taken at 600 nm to estimate cfu/ml of each inoculum from comparisons to OD standard curves, where 0.26 nm = 2.51E + 08 cfu/ml = 1.992 ml inoculation volume. (Supplemental Methodology, Table S5 and Figures S4–S6, Supporting Information). Each representative yeast community was inoculated into the homogenized must with approximately 2.5 × 10^6^ cfu/ml and placed in a 28 ± 1°C room to ferment. This entire process from growing the yeast inoculums, mixing the yeast isolates from each site together to create mixed yeast community inoculums, and inoculating the communities into the Pinot Noir must for fermentation was repeated three times (i.e. three batches). Each vineyard site was represented once in each batch to control for any batch variability. Therefore, there were a total of three replicate ferments per vineyard site for analysis. Malolactic fermentation was neither induced nor suppressed, and metabolites from this process were not measured or analysed.

#### Fermentation monitoring and wine sample collection

Ferments were plunged three successive times daily to submerge the cap and mimic commercial wine-making conditions. Fermentation progress was monitored daily via weight loss (El Haloui et al. 1988). Fermentation was considered complete when ferments had lost a total of more than 5% of their starting weight (El Haloui et al. 1988), or after 10 days of fermentation (whichever came first).

Upon completion, the vessels were plunged to their maximum to press the solids and the liquid was poured into sterile flasks. The flasks sat overnight at 4°C to settle the heavier solids. The wine was then decanted into polypropylene Thermo Scientific Nalgene centrifuge tubes and centrifuged at 6000 *g* in Thermo Scientific Sorvall Lynx4000 Superspeed Centrifuge for 10 minutes to pellet any remaining solids and yeast cells. The supernatant (wine) was transferred to sample containers and stored at −80°C until chemical analysis.

#### Fermentation analysis

Maximum rate of fermentation was determined by taking the derivative of CO_2_, as determined via weight loss, with respect to time dCO_2_/dt (El Haloui et al. 1989). To further examine fermentation kinetics, ethanol by volume (ABV) was measured directly in the final wines using an Anton Parr Alcolyzer Wine M (Table S4, Supporting Information). Residual sugar was measured via the Megazyme ᴅ-fructose and ᴅ-glucose enzymatic assay (Megazyme 2018) (Table S4, Supporting Information). The conversion efficiency of sugar into ethanol was determined via calculation, with an ideal fermentation converting sugar in the following manner: 1*X sugar* (*glucose and fructose*)→2X ethanol + 2*X carbon dioxide*. Prior to fermentation trials, the sterilized must was 22.75° Brix (same across all trials). Therefore, an ideal trial ferment would lose roughly 22 g to CO_2_ production.

### Wine chemical analysis

A total of 28 yeast-derived aroma compounds (esters, higher alcohols, terpenes, C6 alcohols, and fatty acids) were measured using headspace solid-phase microextraction coupled with gas chromatography-mass spectrometry (HS-SPME GC-MS) (Malherbe et al. 2009, Herbst-Johnstone et al. 2013, Pinu et al. 2014, Parish et al. 2016). Each sample was incubated for 10 minutes in the Gerstel MultiPurpose Sampler VT32-20 and agitated at 500 rpm prior to extraction. A 2 cm, 23-guage, 50/30 μm, DVB/CAR/PDMS fibre was exposed to the sample for 60 minutes at 45°C. After extraction, the fibre was transferred to the rear injection port of an Agilent 7890A GC system coupled to a mass selective detector model 5975C inert XL. Helium was used as the carrier gas at a low rate of 1 ml/min. Volatile compounds were separated on a tandem column composed of an Agilent HP-1 ms and an Agilent HP-INNOWax. Agilent MassHunter Quantitative Analysis software was used to quantify the resulting peaks via integration. The integration values were compared to standards to determine the concentration of volatile compounds (μg/l) in each sample.

### Statistical analysis

Contingency tables to investigate if the yeast community composition of the sites differed by region were analysed with chi-square tests using chi-square test calculator (Stangroom 2018), where any zero counts were replaced with 1 to allow the analyses to be conducted; all other analyses were conducted with R via RStudio 3.4.2 (R Studio Team 2020).

To categorize the community composition for each site as a factor for statistical tests against the wine’s chemical composition, a presence/absence method was utilized to form discrete groups based on the yeast species detected. The species present in each community were assigned letters and each community was then given a letter for each member present (Table S1, Supporting Information).

To confirm there were no batch differences between the experimental ferments, the sugar to ethanol conversion efficiency and maximum rate of fermentation were tested using ANOVA (Chambers et al. 1990). The factors of region and community composition were also tested in these analyses.

Because some ferments were incomplete and this may have consequences for the chemical composition of the resulting wines (Conner et al. 1998, Robinson et al. 2009, Mestre et al. 2019), ANOVA was used to test whether the residual sugar concentration varied between regions.

PermANOVA analyses as implemented in the ‘vegan’ package were performed to test the effect of yeast region of origin and community composition on the wines chemical composition and the strata function was implemented to constrain permutations within replicates where applicable (Anderson 2001, Legendre and Legendre 2012, Mcardle and Anderson 2018, Oksanen et al. 2019, R Studio Team 2020). Whether individual aroma compounds varied by yeast region of origin and community composition was analysed with ANOVA and *P-*values were adjusted for multiple tests using the Benjamini and Hochberg method (Benjamini and Hochberg 1995). Constrained correspondence analysis (CCA) was used visualize the data (Legendre and Legendre 2012, Oksanen et al. 2019).

## Results

### Yeast isolation and identification

Yeast community samples were obtained from uninoculated ferments deriving from 17 vineyards when 2° Brix were lost. One sample from Central Otago (CFRP) had lost more than 2° Brix prior to sampling and was subsequently discarded from all further analyses. A total of 1495 isolates were obtained with 432 from Central Otago, 552 from Martinborough, and 511 from Marlborough. In total, 1440 isolates were successfully RFLP profiled and clustered into 13 cohorts. Sanger sequencing indicated these belonged to five taxonomic groups: *S. cerevisiae, Hanseniaspora sp*.,*Metschnikowia sp*.,*Candida zemplinina*, and *S. uvarum*. The *Hanseniaspora species* group contains DNA sequences matching to *H. valbyensis* and *H. uvarum*, and the *Metschnikowia species* group includes *M. pulcherrima* and another *Metschnikowia* sp. not identified to species level (Table 1; Table S2, Supporting Information). Contingency table analyses revealed that the yeast community composition (the numbers of different taxa) significantly differed between the three regions (chi-sq = 346.55, *P* < 1.0 × 10^−05^), confirming the representative yeast communities used to inoculate the lab-scale ferments are regionally distinct.

**Table 1.** Yeast species distribution by region.

	*S. cerevisiae* (%)	*H. uvarum* (%)	*Metschnikowia species* (%)	*S.uvarum* (%)	*C. zemplinina* (%)
Marlborough	21.78	62.97	1.98	10.10	3.17
Martinborough	4.75	80.00	0.40	0.00	14.85
Central Otago	40.70	56.51	2.79	0.00	0.00

### Fermentation

Despite repeated treatments with DMDC, the negative control samples reported the innate yeast community remained viable in the starting juice at approximately 10^3^ cfu/ml: this is 1000 times lower than the 2.5 × 10^6^ cfu/ml inoculation rate of the yeast communities. Weight loss of the control fermentation was an average of two times slower than the inoculated fermentations, but 2 of the 51 inoculated ferments had rate losses slower than the controls (Figures S1–S3, Supporting Information). However, since the same batch of must was used for all experimental ferments, including the controls, any effect of the background community is consistent among all ferments and thus unlikely accounts for any differences between ferments. Fermentation batch had no significant effect on conversion efficiency (ANOVA, *F*_2,39_ = 0.112, *P* = .895), maximum rate of fermentation (ANOVA, *F*_2,39_ = 0.173, *P* = .842), or residual sugar (ANOVA, *F*_2,42_ = 0.61, *P* = .85; Figure S17, Supporting Information).

Yeast community region of origin had a significant impact on conversion efficiency of sugar to ethanol (ANOVA, *F*_2,39_ = 3.74, *P* = .032), and the concentration of residual sugar in the wine (ANOVA, *F*_2,42_ = 3.65, *P* = .035); however, it had no significant effect on the maximum rate of fermentation (ANOVA, *F*_2,39_ = 1.15, *P* = .128). Community composition was found to have a significant impact on conversion efficiency (ANOVA, *F*_6,35_ = 4.87, *P* = 6.88 × 10^−04^), maximum rate of fermentation (ANOVA, *F*_6,35_ = 24.83, *P* = 1.26 × 10^−12^), and the concentration of residual sugar in the wine (ANOVA, *F*_6,30_ = 4.518, *P* = .002).

### Wine chemical analysis

**Figure 2.**
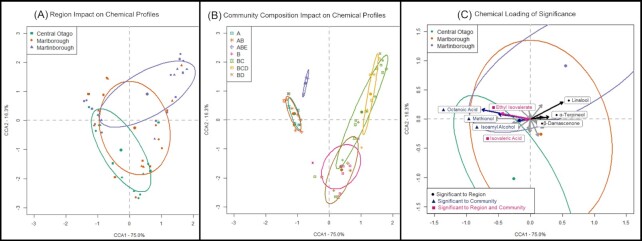
CCA analyses of the experimental ferments. (A) Region of origin impact on aroma compounds coloured by region of origin and depicts 50% ellipses. (B) Community composition impact on aroma compounds coloured by yeast community composition, where A = *S. cerevisiae*, B = *Hanseniaspora species*, C = *Candida zemplinina*, D = *Metschnikowia species*, and E = *S. uvarum*. (C) Region of origin and community composition loadings for aroma compounds according to CCA species score. Vectors representing statistically significant aroma compounds are labelled. Vectors and compounds in black are of significance to region of origin, vectors and compounds in blue to community composition, and vectors in grey are compounds that overlapped those of significance to community and region of origin.

There was a significant effect of yeast community region of origin on wine chemical profiles, with 10% of the variation in wine chemical profiles attributed to yeast community region of origin (PermANOVA, *F*_2,41_ = 3.98, *R*^2^ = 0.106, *P* = .0029, Fig. 2A; Table S3, Supporting Information). Analyses (with error correction incorporated) of each of the 28 compounds showed that 11 significantly differed due to yeast community region of origin (*P*_adj_ range 0.03–1.5 × 10^−7^, Table 2 and Fig. 2B). ANOVA and CCA analyses (Table 2 and Fig. 2C) revealed that β-damascenone, α-terpineol, ethyl isovalerate, isovaleric acid, and linalool differ the most by yeast community region of origin. Tukey HSD reveals these compounds significantly differ between all regions for β-damascenone, linalool, and α-terpineol with concentrations being highest in Martinborough (Figures S7–S9, Supporting Information). For ethyl isovalerate and isovaleric acid concentrations were not significantly different between Central Otago and Marlborough but were significantly higher in these two regions compared with Martinborough (Figures S10 and S11, Supporting Information).

**Table 2.** Volatile aroma compounds measured via HS-SPDE GC-MS. The ions and retention time used to identify each compound are listed along with their statistical significance to community composition and region of origin as determined by ANOVAs run for each compound. All *P*-values have been adjusted using the Benjamini and Hochberg (1995) method. Bold, underlined, and italicized indicate aroma compounds of significance to community composition or region of origin as determined by *P*_adj_ value < .05.

				Community		Region	
	Compound	Ions	Retention times	*F-*value	*P*_adj_ value	*F* value	*P*_adj_ value
Alcohols	1-Butanol	56, 31, 41	11.088	3.19	0.05	* **6.61** *	* **0.03** *
	Benzaldehyde	77, 106, 105	31.25	* **4.56** *	***5.6 × 10−03***	2.13	0.58
	Benzyl alcohol	79, 108, 107	49.326	* **5.71** *	***1.1 × 10−03***	1.35	1.0
	Isoamyl alcohol	55, 42, 70	14.259	* **54.53** *	***3.1 × 10−15***	* **11.88** *	***1.1 × 10−03***
	Isobutanol	43, 41, 74	9.101	* **14.12** *	***8.5 × 10−08***	4.16	0.13
	Methionol	106, 61, 58	40.913	* **83.07** *	***3.1 × 10−15***	* **9.49** *	***4.2 × 10−03***
Esters	Ethyl 2-methyl butanoate	57, 102, 85	13.41	* **18.14** *	***3.1 × 10−09***	0.08	1
	Ethyl butanoate	71, 88, 101	11.105	2.37	0.18	* **6.25** *	* **0.03** *
	Ethyl decanoate	88, 101, 155	51.465	* **10.9** *	***1.9 * × 10−06****	4.86	0.08
	Ethyl hexanoate	88, 99, 101	23.87	* **21.12** *	***3.7 * × 10−10****	3.03	0.29
	Ethyl isobutyrate	43, 71, 116, 88	8.911	2.69	0.106	* **7.31** *	* **0.02** *
	Ethyl isovalerate	88, 85, 115	13.758	* **138.65** *	***3.1 × 10−15***	* **21.63** *	***7.3 × 10−06***
	Ethyl octanoate	88, 101, 127	38.286	* **44.8** *	***3.1 × 10−15***	2.95	0.3
	Ethyl phenylacetate	91, 164	48.127	1.51	0.7	0.2	1.0
	Hexyl acetate	56, 61, 84	25.326	1.02	1.0	3.57	0.21
	Isoamyl acetate	70, 55, 87	15.591	* **9.54** *	***8.4 * × 10−06****	* **9.58** *	***4.2 × 10−03***
	Isobutyl acetate	43, 56, 73	9.896	0.41	1.0	5.69	0.05
	β-phenylethyl acetate	104, 43, 91	49.39	0.93	1.0	0.51	1.0
Terpenes and C-13 norisoprenoids	α-terpineol	59, 93, 121	43.9	* **4.14** *	* **0.01** *	* **25.78** *	***1.4 × 10−06***
	β-damascenone	69, 121, 190	53.521	* **4.97** *	***3.1 × 10−03***	* **32.32** *	***1.5 × 10−07***
	β-ionone	177, 178, 192	59.731	* **13.91** *	***9.3 × 10−08***	0.4	1.0
	Cis–trans rose oxide	139, 69, 83	33.18	* **6.74** *	***3 × 10−04***	3.38	0.23
	Nerol	69, 41, 93, 121	49.442	0.62	1.0	0.26	1.0
	Linalool	71, 93, 121	36.383	* **5.74** *	***1.1 × 10−03***	32.07	1.5 × 10^−07^
Fatty acids	Isobutyric acid	43, 73, 88	33.395	* **14.97** *	***4.2 × 10−08***	1.68	0.83
	Isovaleric acid	60, 87	39.017	* **47.79** *	***3.1 × 10−15***	11.91	1.1 × 10^−03^
	Octanoic acid	60, 73, 101	59.09	* **65.66** *	***3.1 × 10−15***	0.73	1.0
C6 compounds	Hexanol	56, 43, 69	22.645	1.64	0.59	0.72	1.0

There is a significant effect of community composition on wine chemical profiles, explaining 50% of the total variation, 5-fold greater than yeast community region of origin (PermANOVA, *F*_6,41_ = 6.31, *R*^2^ = 0.505, *P* = 9.9 × 10^−5^, Fig. 2B; Table S3, Supporting Information). Figure 2 and Table 2 show the compounds that differed due to yeast community composition, and as well as ethyl isovalerate, isovaleric acid, which also differed by yeast community region of origin, isoamyl alcohol, methionol, and octanoic acid differed the most between ferments with different yeast community compositions. Tukey HSD reveals these compounds significantly differ between communities with *Saccharomyces* species present and those without *Saccharomyces* species present. For all these compounds, concentrations were higher if *S. cerevisiae* was present in the yeast community (Figures S12–S16, Supporting Information). Fermentation batch, and various interactions between factors had no significant effect on wine chemical profiles (Table S3, Supporting Information).

## Discussion

The data and analyses presented here provides evidence that region-specific mixed yeast communities contribute to the regional distinctiveness of a wine’s volatile composition, providing the first objective evidence that microbial communities, beyond the fermenting yeast *S. cerevisiae*, have the potential to contribute to a regional wine distinctiveness, or *terroir*. In fact, the 10% difference in wine chemistry due to regional yeast communities observed here is consistent with that reported for the effect of regionally genetically distinct populations of *S. cerevisiae* on Sauvignon Blanc (Knight et al. 2015). Additionally, a novel method to prepare and inoculate mixed-yeast communities for fermentation trials in a controlled, replicable manner is detailed.

As first formulated, the hypothesis concerning whether there is a microbial aspect to *terroir* did not claim that microbes played a dominate role in regional wine differentiation (Gayevskiy and Goddard 2012), but simply tested whether microbes may play any role. The data are converging to suggest that microbes do play a role, but that this is small and just one part of the many other factors that drive wine regional distinctness, which makes intuitive sense. The salient point is that regionally differentiated microbes do play a part in the complex drivers of wine regionality. As seen in *S. cerevisiae*, it is possible different non-*Saccharomyces* species of yeast have genetically distinct regional subpopulations (Knight and Goddard 2015, Alexandre 2020), which could potentially be contributing to the regional wine differences observed here. This highlights the importance of understanding yeast community differentiation at a finer scale of strain distinctiveness when considering how these mixed communities contribute to regional wine characteristics. Further investigation into the strain differences of the isolates used in this study is required to verify if this is the source of the variation observed here.

Wines from yeast communities isolated from Central Otago and Martinborough have greater separation between them with Marlborough resting in the middle (Fig. 2A). This is consistent with patterns in wine chemistry reported for *S. cerevisiae* (Knight et al. 2015) and with differentiation in microbial communities associated with vines and wines in New Zealand generally (Taylor et al. 2014, Knight and Goddard 2015, Morrison-Whittle and Goddard 2015). Marlborough is a major hub for the New Zealand wine industry, accounting for 71% of New Zealand’s wine producing area compared to 3% for Martinborough and 5% for Central Otago (New Zealand Wine Growers 2022b). This increased industry activity and transportation of fruit from smaller regions into Marlborough for fermentation can facilitate yeast dispersal amongst geographic locations via human assisted migration (Liti et al. 2009, Goddard et al. 2010, Knight et al. 2015, Liti 2015). Therefore, it is plausible that the overlap observed between Marlborough and the other regions could be explained, in part, by human assisted migration, but further investigation is required. This pattern also mirrors that of geographic space, with Marlborough physically located between Martinborough and Central Otago. It may be that the yeast communities become more dissimilar with increasing geographic distance, and this is then reflected in the chemical differentiation in the wines. Previous research in New Zealand Sauvignon Blanc vineyards found that the geographic distance separating microbial communities explained 6.1% of the variance in community composition observed (Morrison-Whittle and Goddard 2015). A Chilean study also found that dissimilarities amongst leaf and berry fungal communities increased with geographic distance (Miura et al. 2017). More extensive sampling of additional regions would be required to objectively test this for Pinot Noir in New Zealand.

Ethyl octanoate, isoamyl acetate, isoamyl alcohol, methionol, linalool, β-damascenone, ethyl isobutyrate, ethyl isovalerate, ethyl-2-methyl butanoate, isovaleric acid, and isobutyric acid have been reported as being significant to Pinot Noir aroma around the world (Brander et al. 1980, Miranda-Lopez et al. 1992, Fang and Qian 2006, Rutan et al. 2014). This study adds α-terpineol, 1-butanol, and ethyl butanoate as being important to regional distinctiveness of New Zealand Pinot Noir. Exactly how the chemical composition of red wines contribute to the sensory perception of different characteristics is complex and poorly understood in red wines; however, these compounds of significance are reported to contribute to Pinot Noir sensory properties in a variety of ways. Esters contribute fruity aromas to wine with ethyl isobutyrate attributed to strawberry, ethyl isovalerate to cherry, ethyl-2-methyl butanoate to fruit and resin, isoamyl acetate and ethyl butanoate both to fruit, and ethyl octanoate to baked fruit aromas in Pinot Noir (Fang and Qian 2006, Rutan et al. 2014). Other studies suggest that ethyl octanoate increases the perception of cherry aroma in Pinot Noir, but when in combination with 2-phenyl ethanol it increased the violet aroma (Tomasino et al. 2015). Savoury aromas can be attributed to alcohols with methionol responsible for vegetable and potato and isoamyl alcohol for cheese and overripe banana aromas in Pinot Noir (Rutan et al. 2014). Terpenes and norisoprenoids, such as linalool, α-terpineol, and β-damascenone have floral and fruity aromas with linalool contributing floral, α-terpineol sweet floral, and β-damascenone tea, floral, fruity, and honey aromas (Fang and Qian 2006, Rutan et al. 2014). Monoterpenes have also been suggested to have an indirect effect by enhancing or suppressing Pinot Noir wine attributes, rather than contributing directly to them (Longo et al. 2021). Isovaleric acid and isobutyric acid are fatty acids that both have cheese aromas (Rutan et al. 2014). The complexity of how compounds may be perceived in wine means we can only speculate on the differences these compounds contribute to regional Pinot Noir aroma and flavour and controlled sensory trials are required to confirm any differences in perception.

While this research adds to our understanding of the contribution of mixed yeast communities to regional wine attributes, the ability of microbes to contribute to regional wine attributes is possibly larger than that reported here. First, the diversity of microorganism in wine fermentations is larger than just yeasts (Povhe Jemec et al. 2001, Selli et al. 2005, Goddard 2008, Zott et al. 2008, Šuranská et al. 2016, Bagheri et al. 2017). As few as five different species of yeast are reported here, while there are numerous other species of yeast known to contribute to wine fermentation. The low diversity of species found in this study could be a result of the limited number of isolates we could manage while using a culturing approach; however, this approach was necessary to be able to replicate and test the mixed yeast communities in experimental ferments. Despite this, grape juice is a hostile environment with low pH and a high osmotic pressure, and previous studies have also reported low yeast diversity during fermentation (e.g. Goddard 2008). Additionally, bacteria may also influence regional wine characteristics (Bokulich et al. 2016); however, this has not yet been tested in a controlled environment or with mixed bacterial communities. Similar methodologies to those used in this study could be utilized to explore whether bacterial communities also contribute to regional character and could potentially shed light on how these natural isolates impact both alcoholic fermentation and malolactic fermentation in red and white wines. Second, this study does not consider how microbial communities present in the vineyard may influence grape production and quality throughout the growing season. Different geographic regions experience different microbial disease pressures, but how fungi and bacteria affect fruit development in other ways is not well-understood. For example, *Botrytis cinerea* and other grapevine pathogens have long-lasting effects on grape development in the vineyard, which impacts wine quality (Barata et al. 2012, Blanco-Ulate et al. 2017, Griggs et al. 2021). This additional information would give a more complete picture of how microbial communities (inclusive of yeast and bacteria) contribute to regional character and warrants further investigation to test such hypotheses.

There are two main caveats to this study: first, the Pinot Noir juice used for the experimental ferments could not be completely sterilized prior to inoculation. As such, the negative controls did eventually ferment during the trials. Statistical analyses of these control samples compared with our experimental ferments shows they fermented slower, indicating our inoculated yeast communities were active and outgrowing any ambient microbial communities in the must. Since the same batch of must was used for all experimental ferments, including the controls, any effect of the background community is consistent among all ferments and thus unlikely accounts for any differences between treatments (i.e. this is not confounded to one region), and thus the regional distinctions we detected were indeed due to differences in the regional yeast communities inoculated. Second, not all ferments finished, and some had high levels of residual sugar. Statistical analyses report the residual sugar in the wines varied with yeast community region of origin, potentially confounding the results of regional differentiation in wine chemistry. However, the competency of regional yeast communities to complete fermentation is a function of the species composition of those ferments; and given the yeast communities are regionally distinct, it could be argued that residual sugar (and by proxy ferment completeness) is a function of the microbial community, which is what we aimed to test. Furthermore, yeast-derived aroma compounds are generated throughout all stages of fermentation (Swiegers and Pretorius 2005, Hall et al. 2017), such as thiols, which are primarily generated during early stages of fermentation by non-*S. cerevisiae* yeast (Zott et al. 2011).

The use of regionally distinct ‘native’ microbes in fermentation is of increasing interest to the wine industry and in other fermentation products. Currently, this is only possible via spontaneous fermentation, which carries risks of spoilage and incomplete fermentation. This work describes a method of creating and using representative region-specific synthetic yeast communities for wine fermentation, i.e. reproducible and effective. If scaled-up, this method provides significant potential to produce tools for winemakers to safely use the region-specific natural microbial biodiversity inherent to their sites to add distinctness and value to products. Furthermore, the approach used in this study could be leveraged to experimentally test similar mixed microbial ecologies beyond those found during wine fermentations. Overall, this work highlights the importance, both economically and ecologically, of better understanding the origins and maintenance of microbial diversity to promote sustainable management practices that protect and potentially enhance these local communities.

## Supplementary Material

foad005_Supplemental_File
