# Supplementary material for: Mixed yeast communities contribute to regionally distinct wine attributes
Source: FEMS Yeast Res. 2023 Feb 1;23:foad005. doi: 10.1093/femsyr/foad005 (PMC9952052; doi:10.1093/femsyr/foad005)
Supplement: foad005_Supplemental_File [file foad005_supplemental_file.docx]

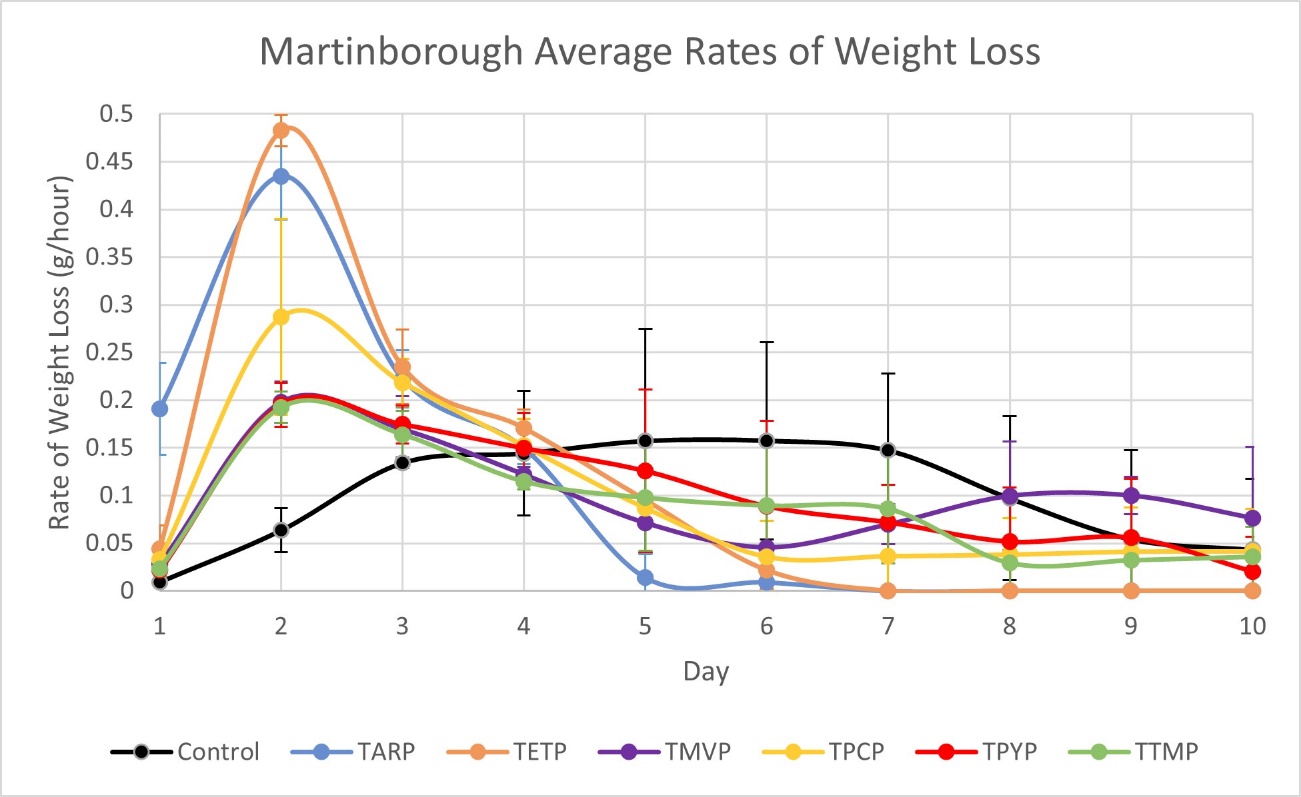


**Supplemental Figure 1:** Martinborough average rates of weight loss. Daily fermentation rates have been averaged across the three trials. Standard deviation between the trials is indicated by the error bars


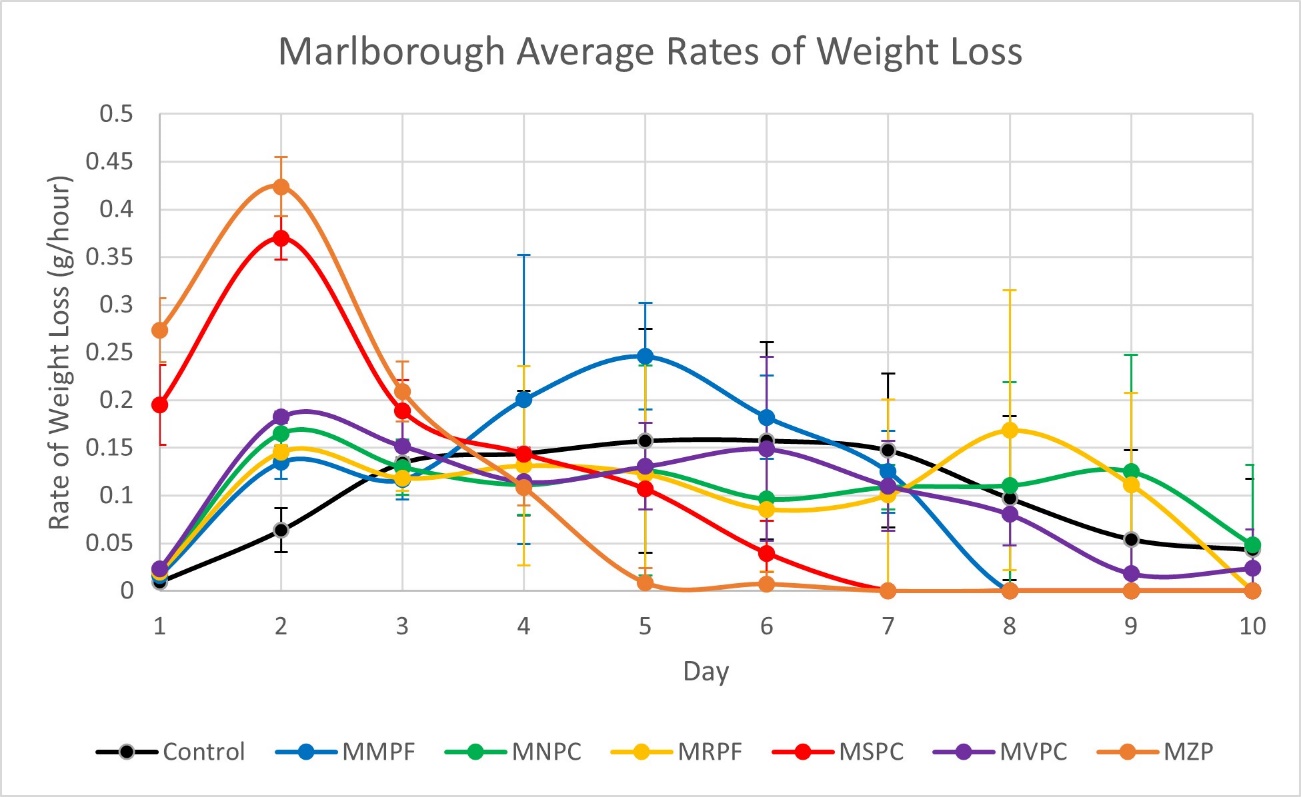


**Supplemental Figure 2:** Marlborough average rates of weight loss. Daily fermentation rates have been averaged across the three trials. Standard deviation between the trials is indicated by the error bars


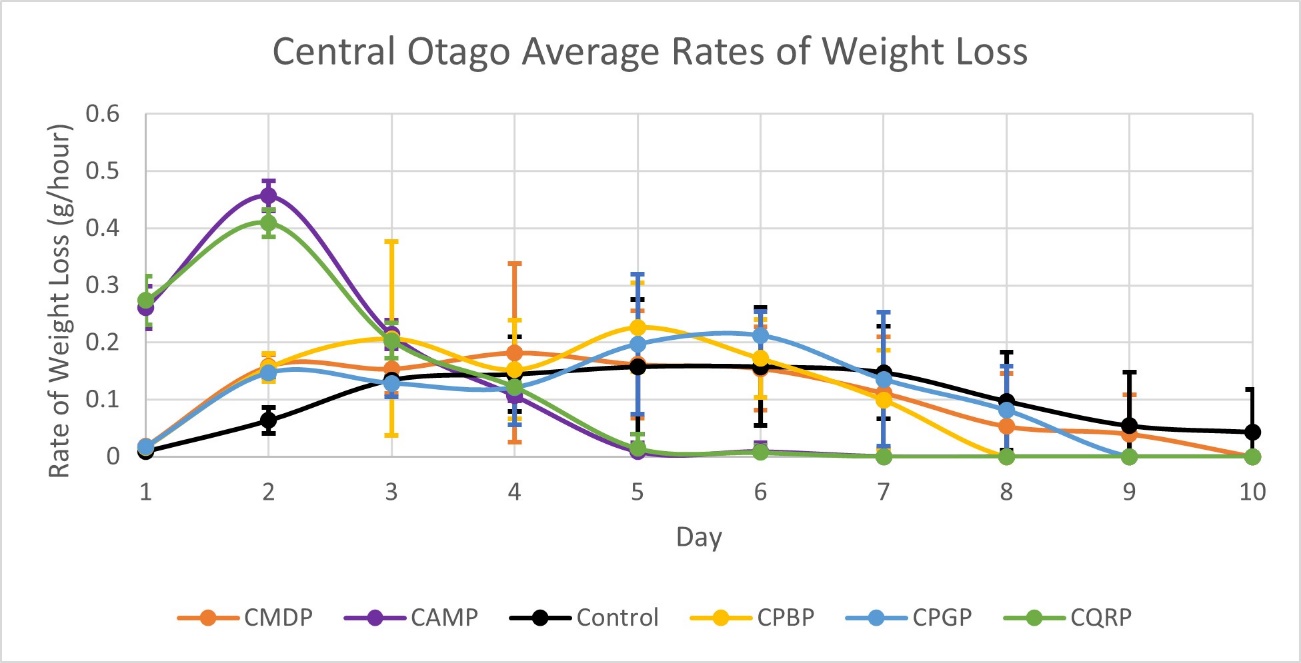


**Supplemental Figure 3****:** Central Otago average rates of weight loss. Daily fermentation rates have been averaged across the three trials. Standard deviation between the trials is indicated by the error bars


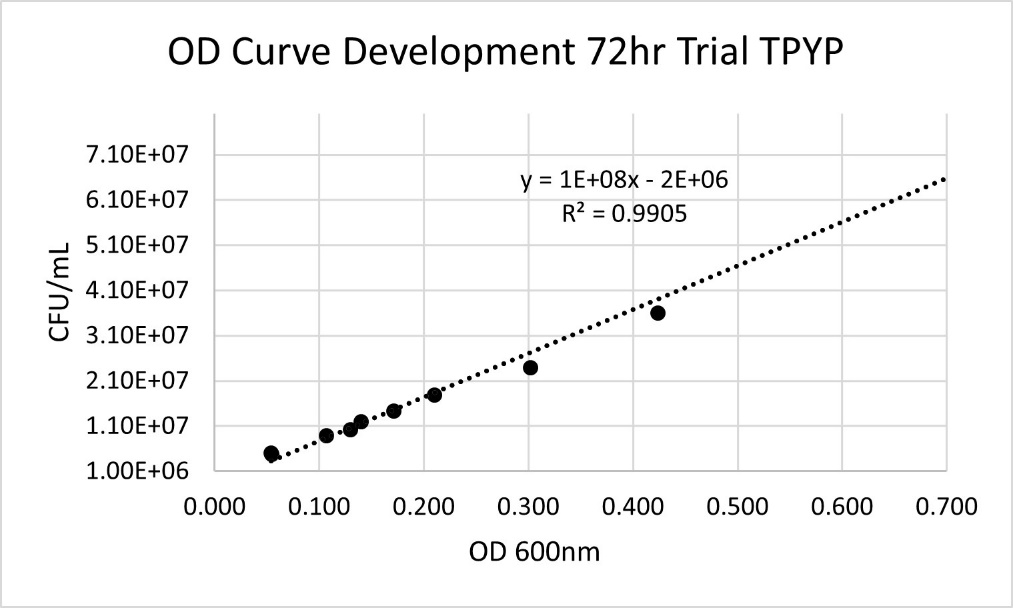


**Supplemental Figure 4:** Optical Density Curve for TPYP after 72 Hours of growth


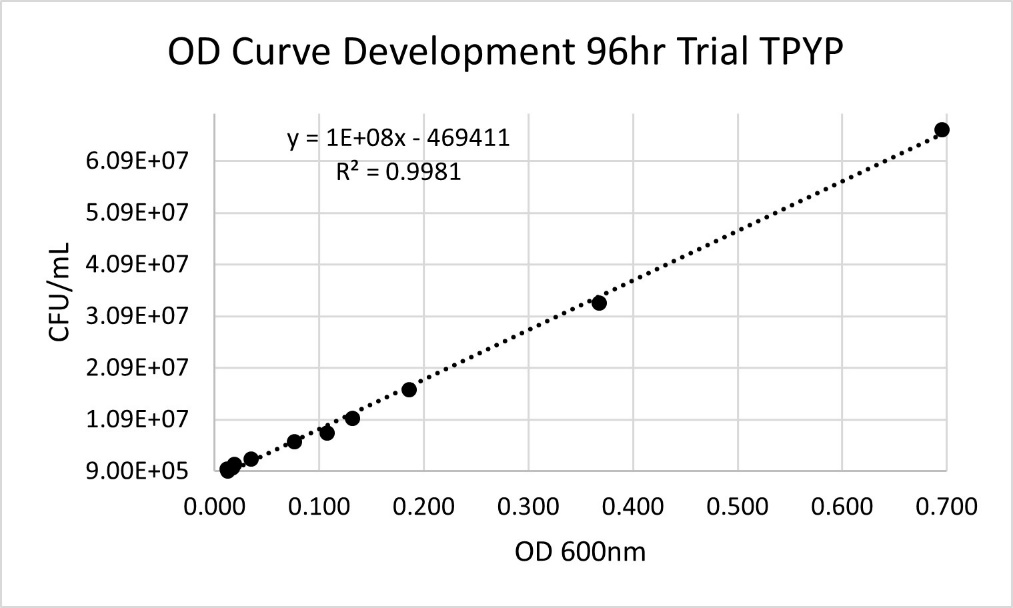


**Supplemental Figure 5:** Optical density curve for TPYP after 96 hours of growth


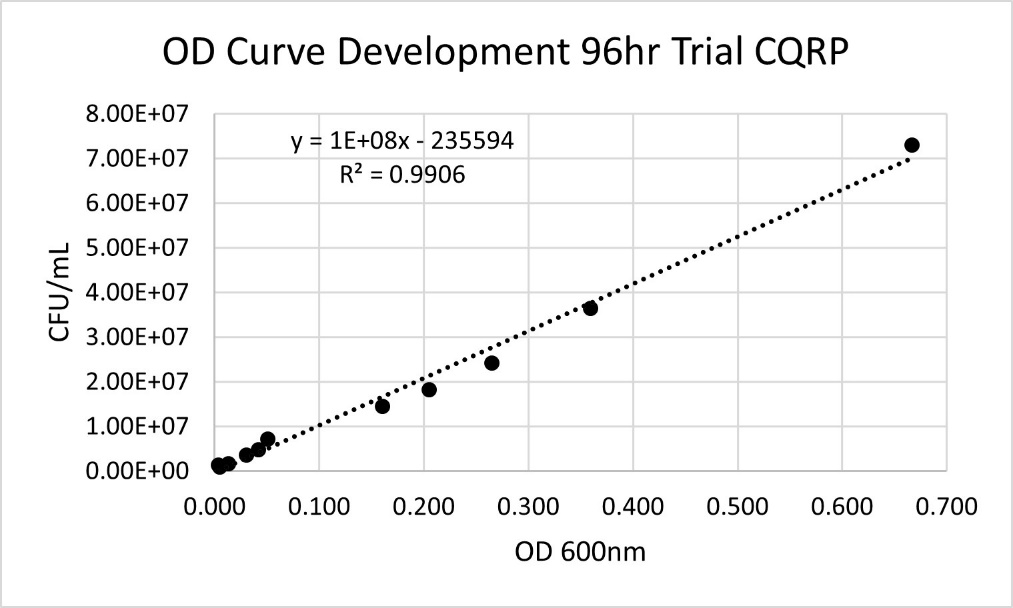


**Supplemental Figure 6:** Optical density curve for CQRP after 96 hours of growth


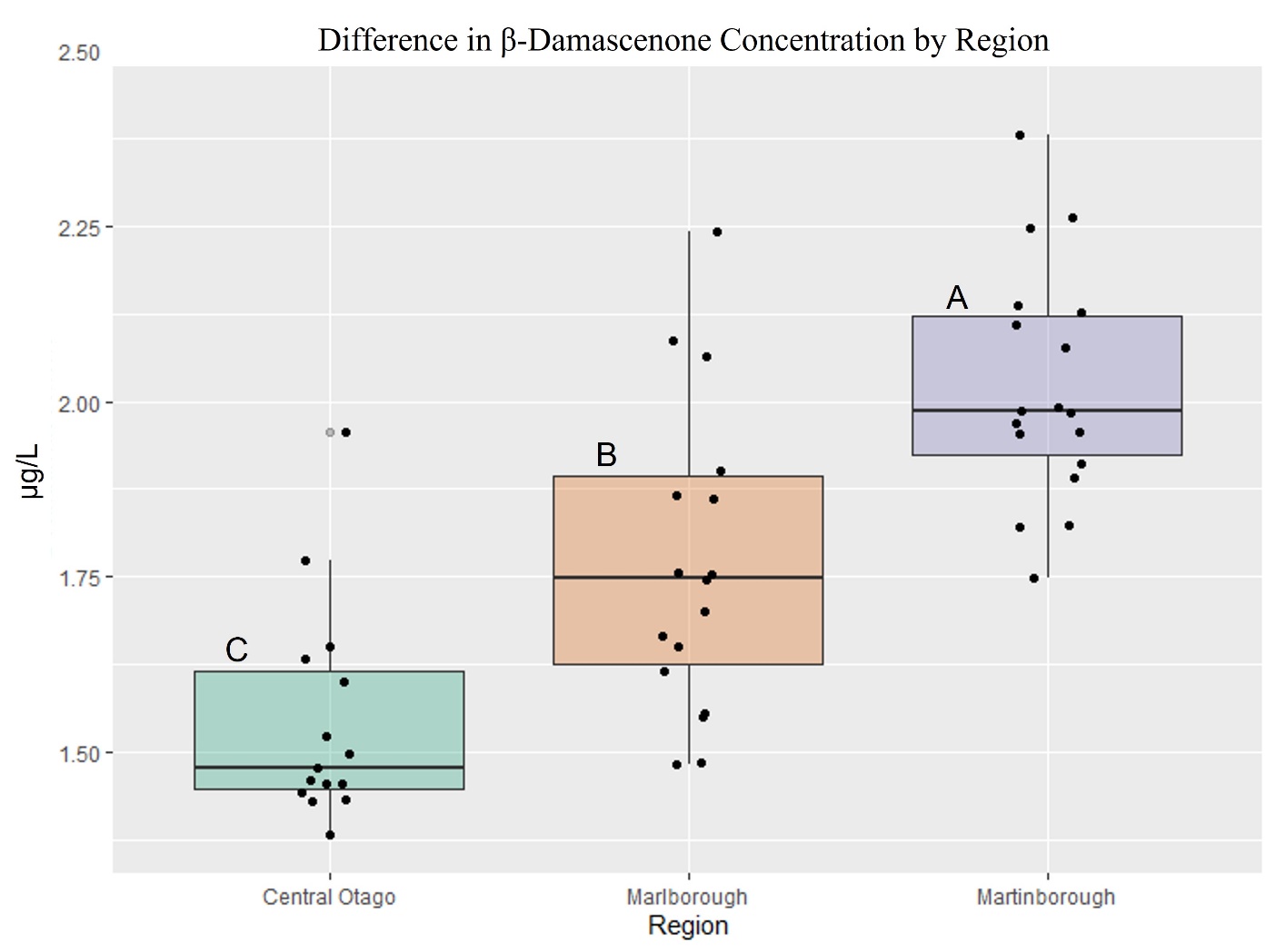


**Supplemental Figure 7:** Difference in β-Damascenone Concentration by Region


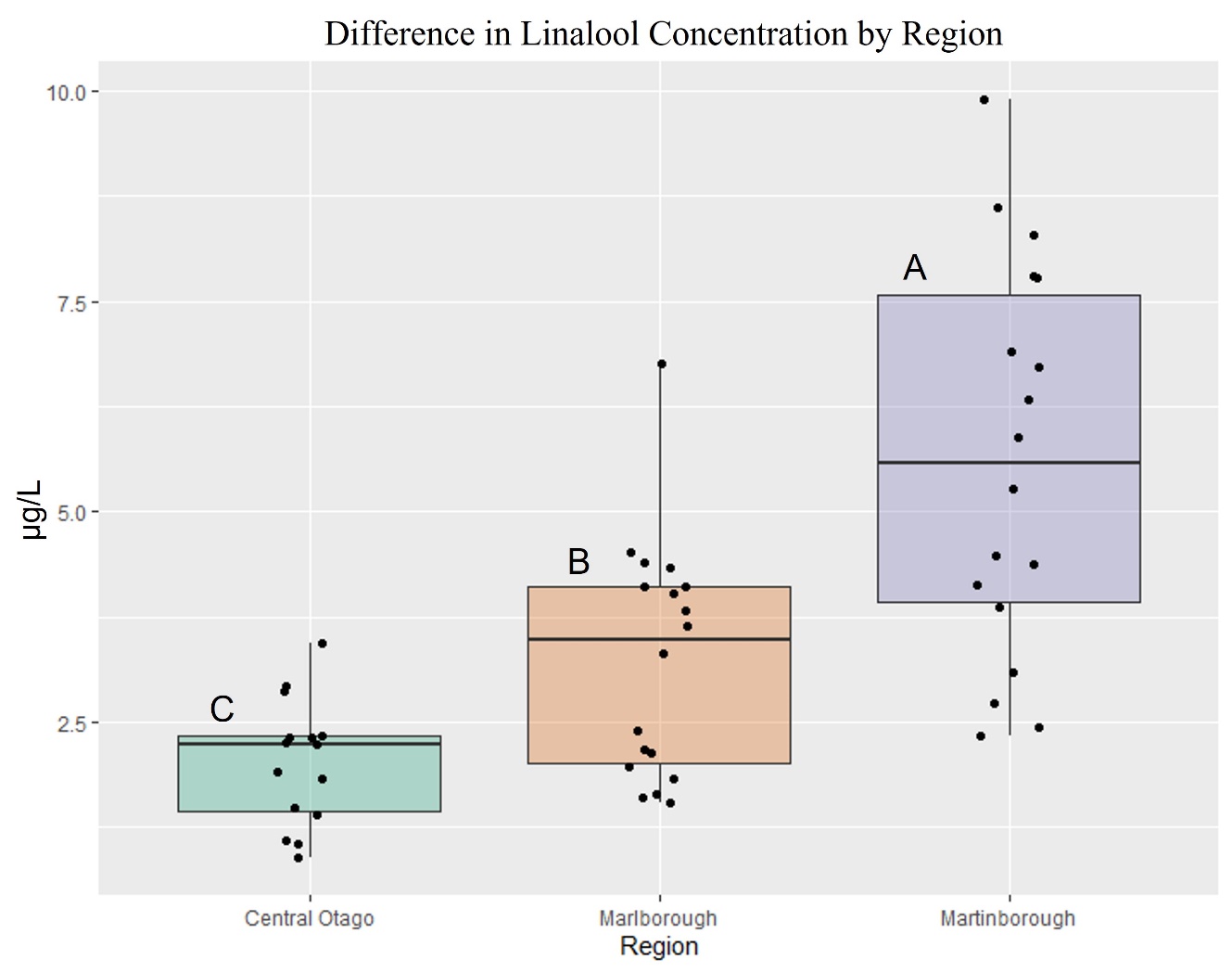


**Supplemental Figure 8:** Difference in Linalool Concentration by Region


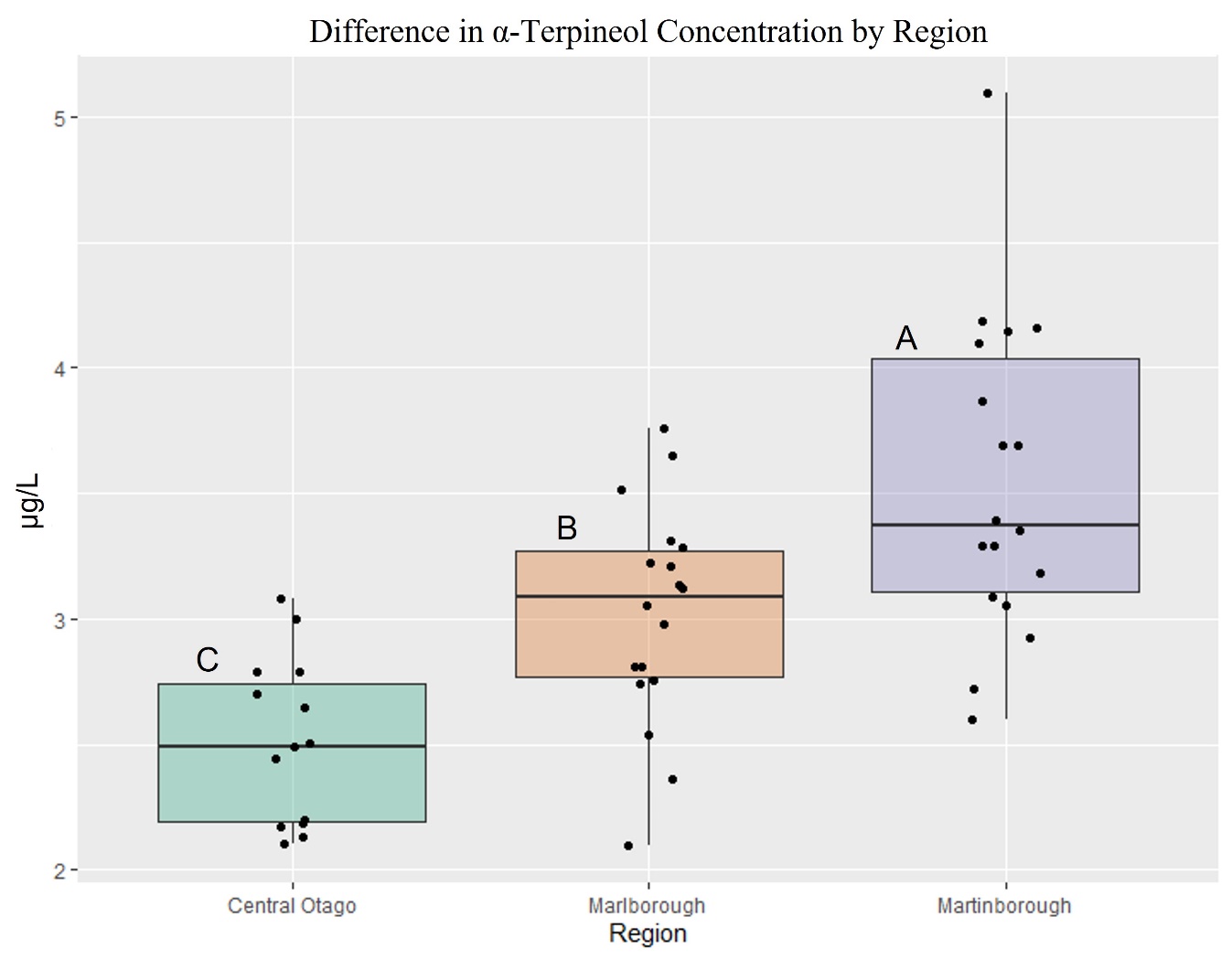


**Supplemental Figure 9:** Difference in α-Terpineol Concentration by Region


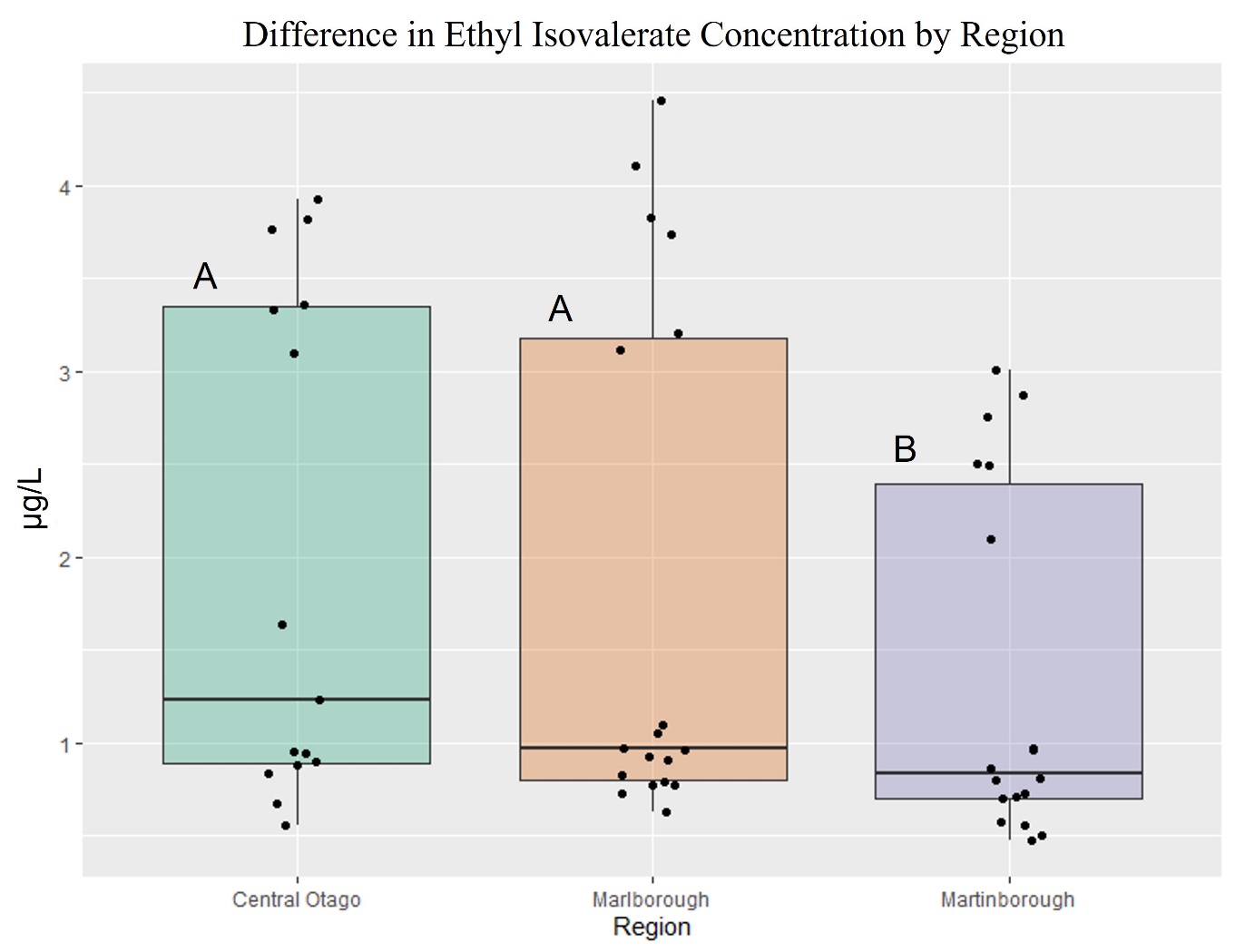


**Supplemental Figure 10:** Difference in Ethyl Isovalerate Concentration by Region


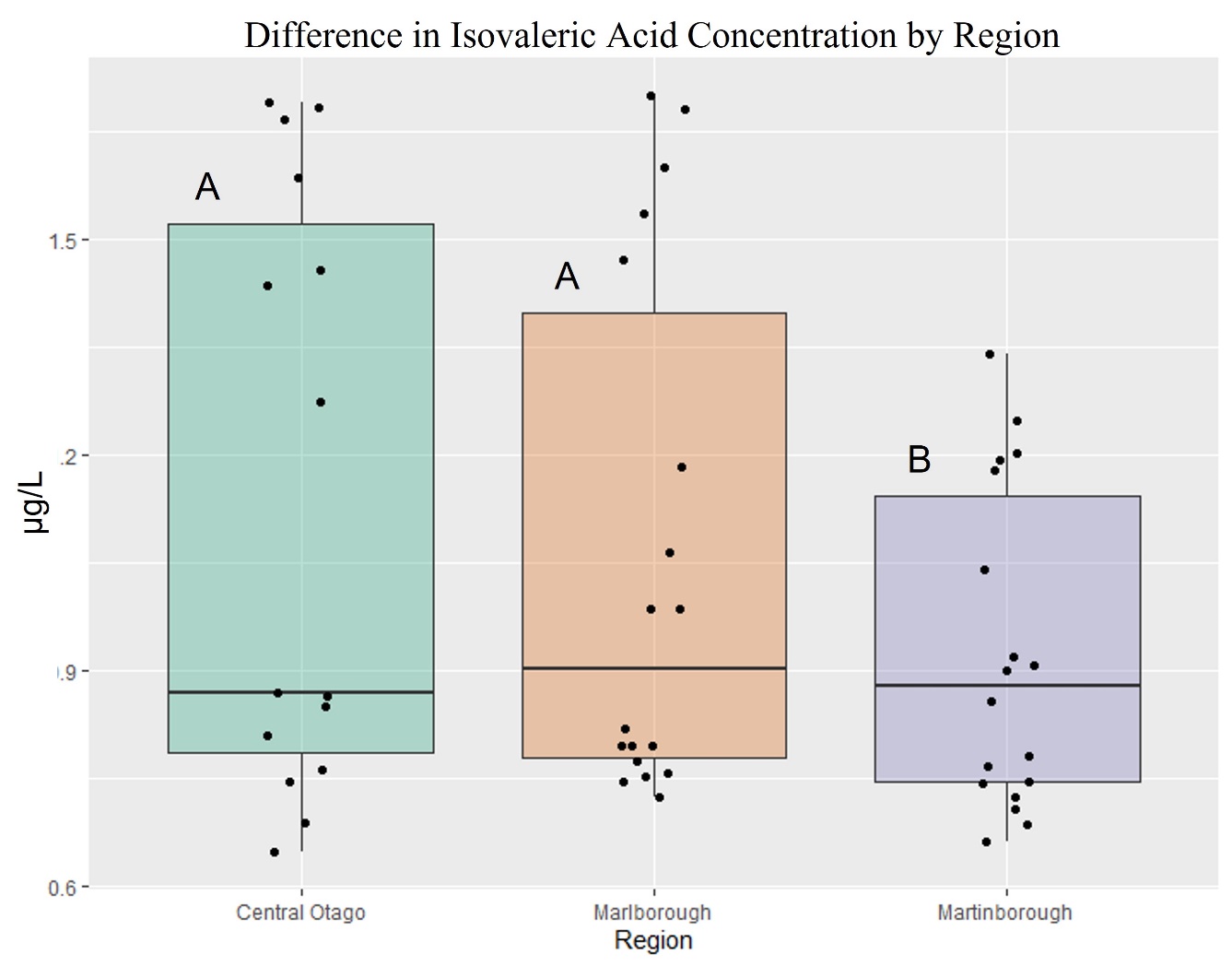


**Supplemental Figure 11:** Difference in Isovaleric Acid Concentration by Region


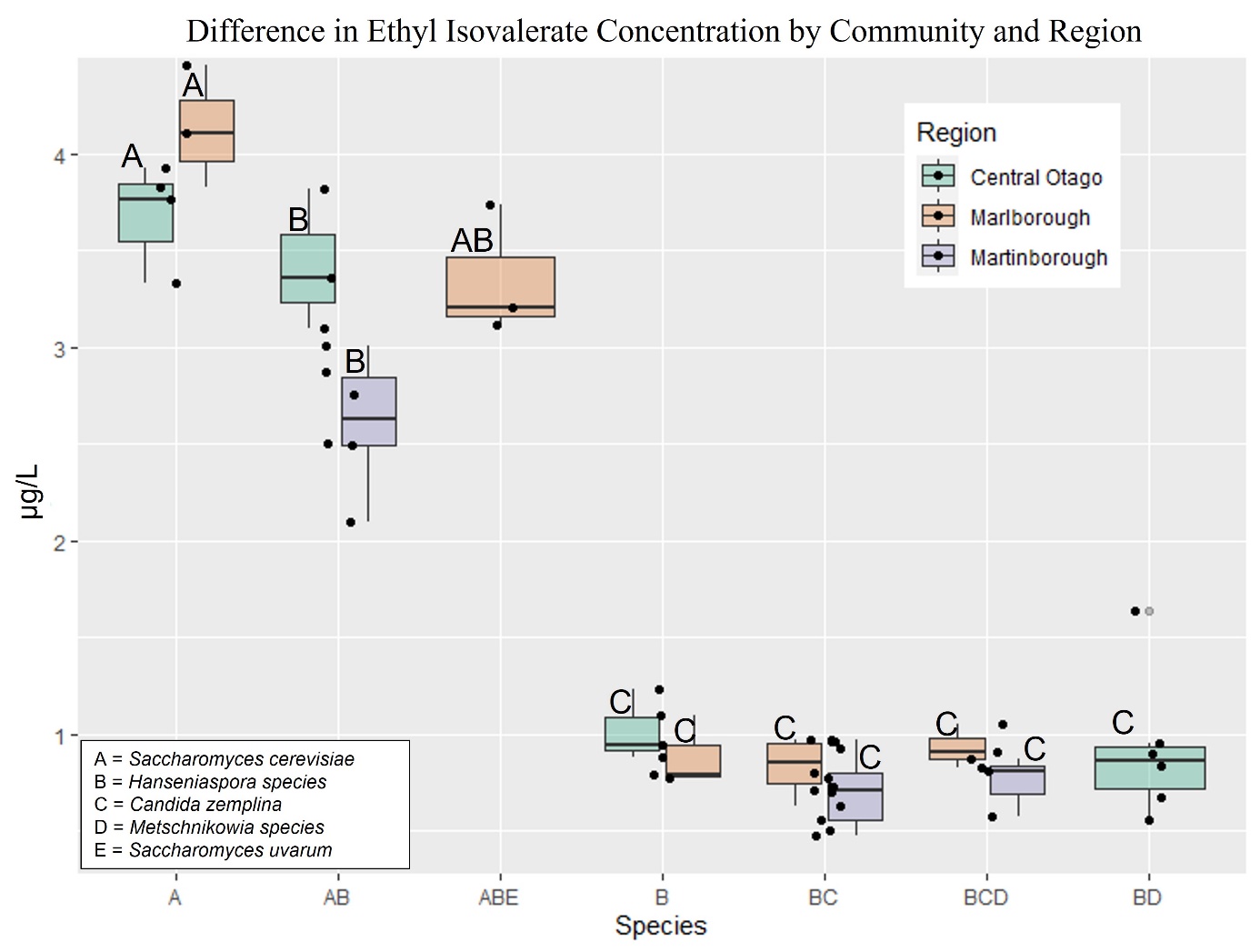


**Supplemental Figure 12:** Difference in Ethyl Isovalerate Concentration by Community and Region


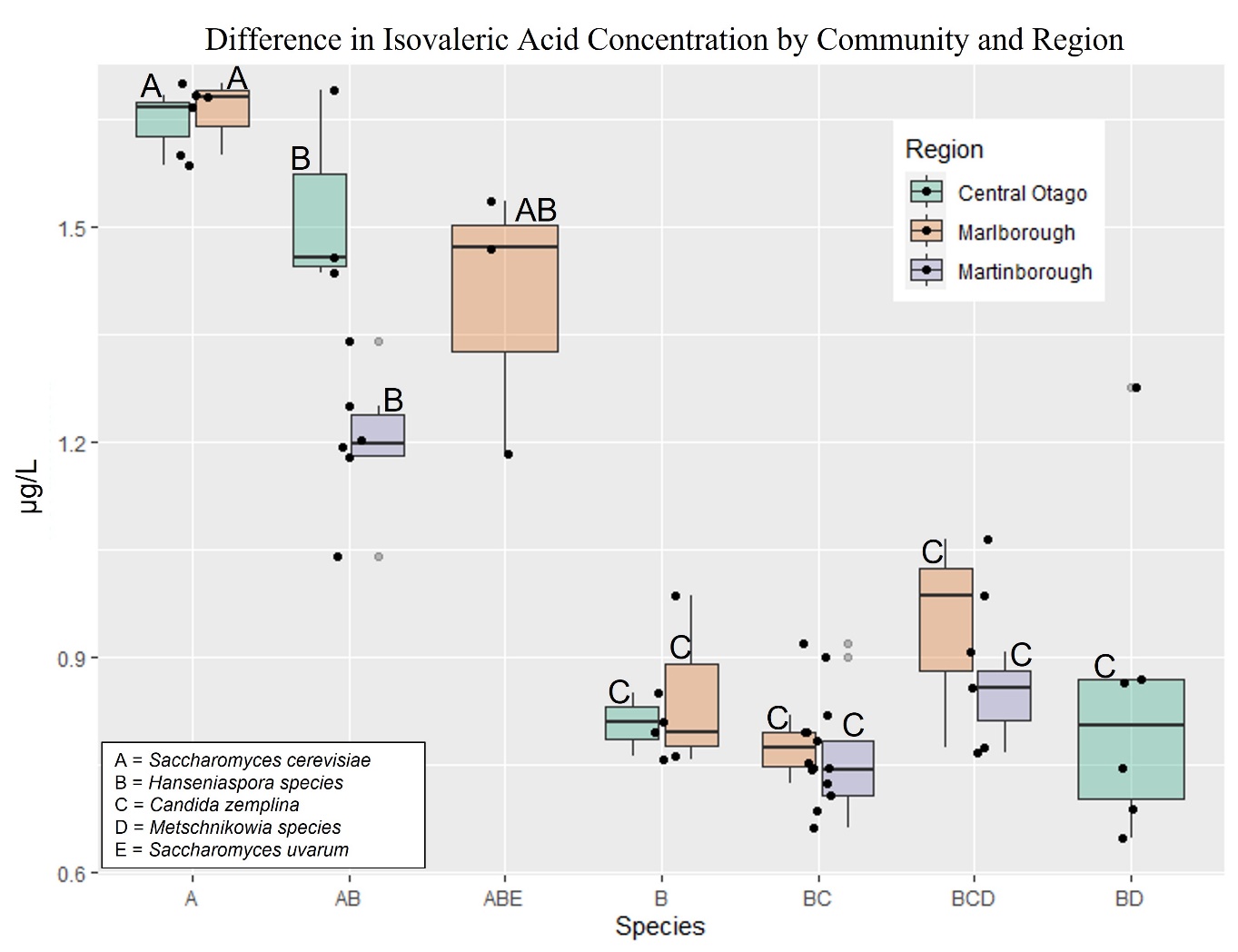


**Supplemental Figure 13:** Difference in Isovaleric Acid Concentration by Community and Region


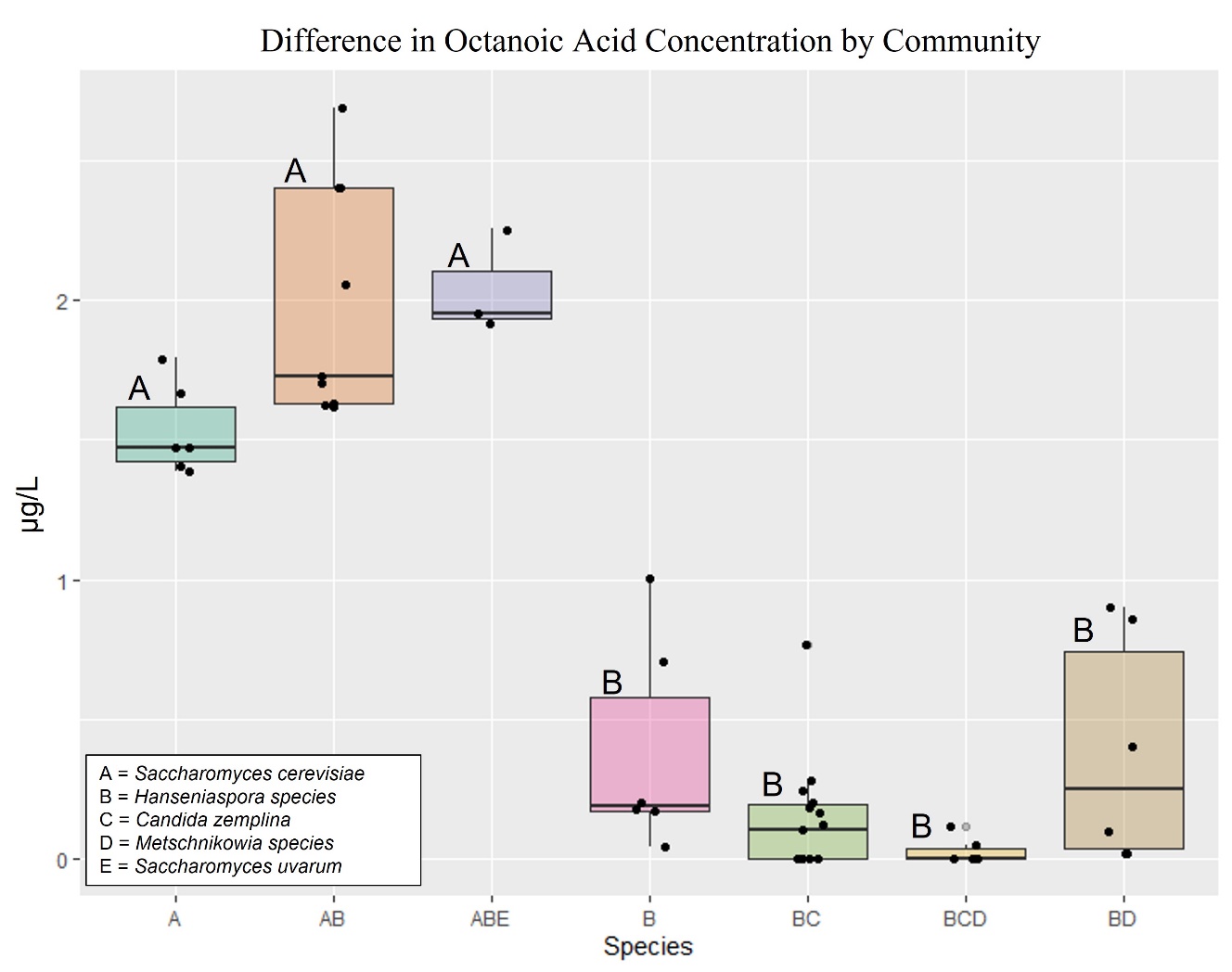


**Supplemental Figure 14:** Difference in Octanoic Acid Concentration by Community


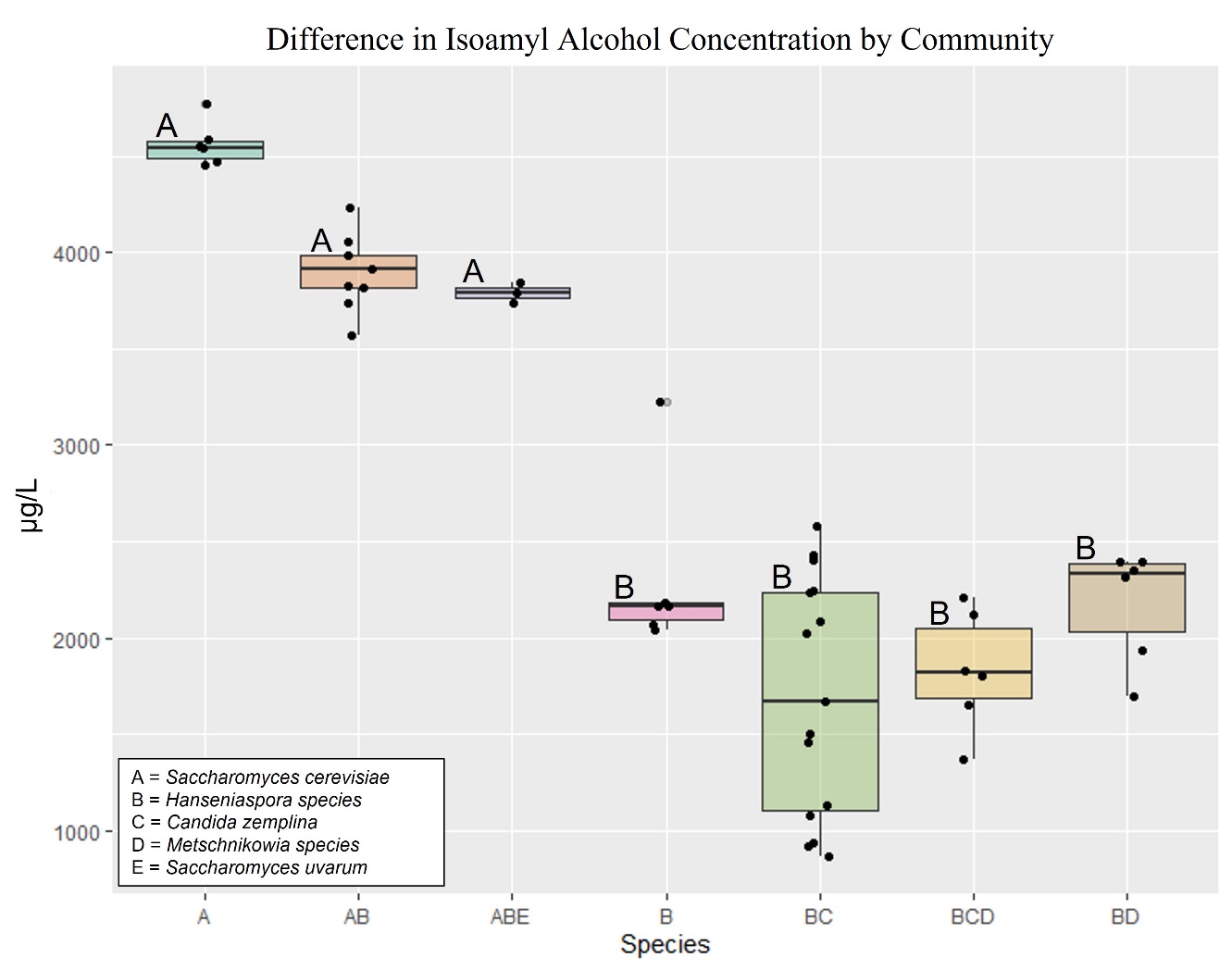


**Supplemental Figure 15:** Difference in Isoamyl Alcohol Concentration by Community


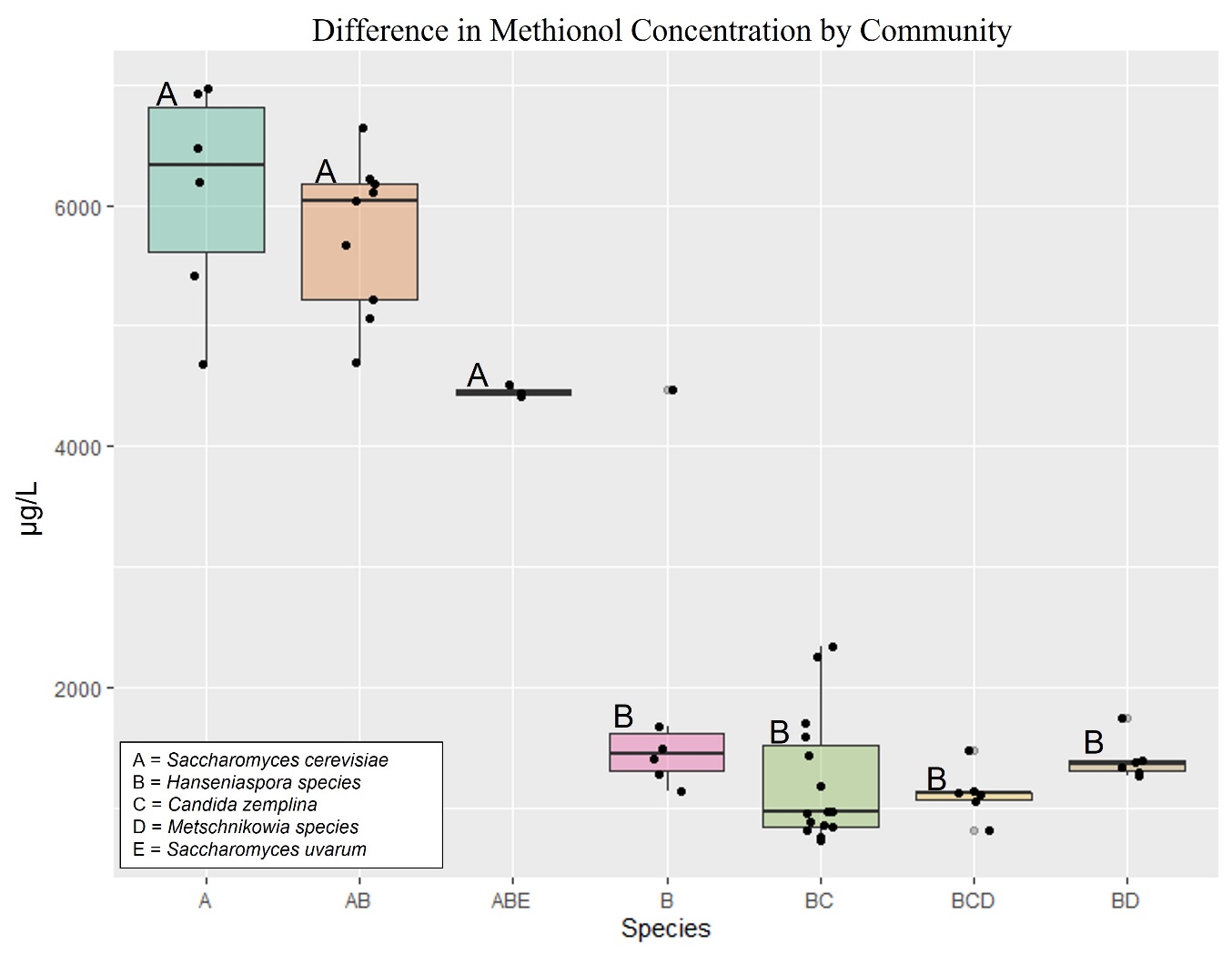


**Supplemental Figure 16:** Difference in Methionol Concentration by Community


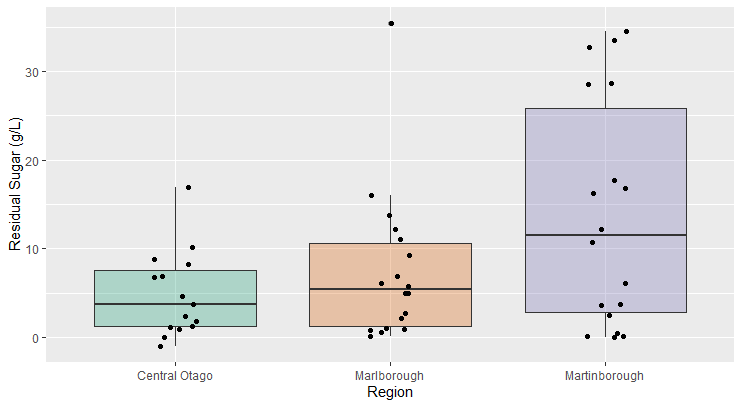


**Supplemental Figure 17:** Residual Sugar by Region

**Supplemental Table 1:** Community composition factor representation summary. A = *Saccharomyces cerevisiae*, B = *Hanseniaspora species*, C = *Candida zemplinina*, D = *Metschnikowia species*, and E = *Saccharomyces uvarum*.

| Region | Sample | Community Composition | Code |
| --- | --- | --- | --- |
| **Marlborough** | MZP | *S. cerevisiae* | A |
|  | MMPF | *H. species, C. zemplinina* | BC |
|  | MNPC | *H. species, M. species, C. zemplinina* | BCD |
|  | MRPF | *H. species* | B |
|  | MSPC | *S. cerevisiae, H. species, S. Uvarum* | ABE |
|  | MVPC | *H. species, C. zemplinina* | BC |
| **Martinborough** | TARP | *S. cerevisiae, H.species* | AB |
|  | TPCP | *H. species, C. zemplinina* | BC |
|  | TETP | *S. cerevisiae, H. species* | AB |
|  | TMVP | *H. species, M. species, C. zemplinina* | BCD |
|  | TTMP | *H. species, C. zemplinina* | BC |
|  | TPYP | *H. species, C. zemplinina* | BC |
| **Central Otago** | CQRP | *S. cerevisiae* | A |
|  | CAMP | *S. cerevisiae, H. species* | AB |
|  | CPBP | *H. species* | B |
|  | CPGP | *H. species, M. species* | BD |
|  | CMDP | *H. species, M. species* | BD |

**Supplemental Table 2:** Distribution of yeast species based on proportion of total isolates identified for each sample

| **Region** | **Sample** | ***Saccharomyces cerevisiae*** | ***Hanseniaspora species*** | ***Metschnikowia species*** | ***Saccharomyces uvarum*** | ***Candida zemplinina*** |
| --- | --- | --- | --- | --- | --- | --- |
| Marlborough | MZP | 100% | - | - | - | - |
|  | MMPF | - | 99% | - | - | 1% |
|  | MNPC | - | 85% | 11% | - | 3% |
|  | MRPF | - | 100% | - | - | - |
|  | MSPC | 21% | 23% | - | 56% | - |
|  | MVPC | - | 85% | - | - | 15% |
| Martinborough | TARP | 27% | 73% | - | - | - |
|  | TPCP | - | 66% | - | - | 34% |
|  | TETP | 1% | 99% | - | - | - |
|  | TMVP | - | 62% | 2% | - | 36% |
|  | TTMP | - | 89% | - | - | 11% |
|  | TPYP |  | 97% | - | - | 3% |
| Central Otago | CQRP | 100% | - | - | - | - |
|  | CAMP | 99% | 1% | - | - | - |
|  | CPBP | - | 100% | - | - | - |
|  | CPGP | - | 95% | 5% | - | - |
|  | CMDP | - | 91% | 9% | - | - |

**Supplemental Table 3:** PERMANOVA analysis testing 28 aroma compounds against the three factors of community composition, the region each community originated from, and trial were performed. Region of Origin X Community X Trial is shown here.

|  | Degrees of Freedom | R^2^ | F | P Value |
| --- | --- | --- | --- | --- |
| Region | 2 | 0.106 | 3.98 | 2.9x10^-03^ |
| Community | 6 | 0.505 | 6.31 | 9.99x10^-05^ |
| Trial | 2 | 0.034 | 1.27 | 0.264 |
| Region:Community | 2 | 0.062 | 2.31 | 0.032 |
| Region:Trial | 4 | 0.034 | 0.644 | 0.838 |
| Community:Trial | 12 | 0.105 | 0.658 | 0.955 |
| Region:Community:Trial | 4 | 0.035 | 0.649 | 0.858 |
| Residual | 9 | 0.120 |  |  |
| Total | 41 | 1.00 |  |  |

**Supplemental Table 4:** Residual sugar and ethanol (% v/v) for all trials

| Sample | Region | Vineyard | Ethanol (% v/v) | Residual sugar (g/L) |
| --- | --- | --- | --- | --- |
| CAMP1 | Central Otago | CAMP | 12.99 | 0.04 |
| CAMP2 | Central Otago | CAMP | 13.33 | 1.29 |
| CAMP3 | Central Otago | CAMP | 13.21 | 0.85 |
| CMDP1 | Central Otago | CMDP | 12.16 | 6.89 |
| CMDP2 | Central Otago | CMDP | 12.35 | 8.83 |
| CMDP3 | Central Otago | CMDP | 12.56 | 6.81 |
| CPBP1 | Central Otago | CPBP | 13.39 | 1.09 |
| CPBP2 | Central Otago | CPBP | 12.5 | 10.2 |
| CPBP3 | Central Otago | CPBP | 12.88 | 8.25 |
| CPGP1 | Central Otago | CPGP | 12.86 | 3.69 |
| CPGP2 | Central Otago | CPGP | 12.79 | 4.59 |
| CPGP3 | Central Otago | CPGP | 12.12 | 16.88 |
| CQRP1 | Central Otago | CQRP | 13.32 | -0.99 |
| CQRP2 | Central Otago | CQRP | 13.36 | 2.34 |
| CQRP3 | Central Otago | CQRP | 13.47 | 1.82 |
| MMPF1 | Marlborough | MMPF | 13.19 | 0.58 |
| MMPF2 | Marlborough | MMPF | 12.52 | 11.08 |
| MMPF3 | Marlborough | MMPF | 12.71 | 6.89 |
| MNPC1 | Marlborough | MNPC | 10.88 | 16.01 |
| MNPC2 | Marlborough | MNPC | 12.8 | 4.94 |
| MNPC3 | Marlborough | MNPC | 11.22 | 13.72 |
| MRPF1 | Marlborough | MRPF | 12.45 | 5.78 |
| MRPF2 | Marlborough | MRPF | 11.42 | 12.2 |
| MRPF3 | Marlborough | MRPF | 12.57 | 9.2 |
| MSPC1 | Marlborough | MSPC | 12.87 | 2.7 |
| MSPC2 | Marlborough | MSPC | 12.75 | 2.15 |
| MSPC3 | Marlborough | MSPC | 12.51 | 4.94 |
| MVPC1 | Marlborough | MVPC | 13.2 | 0.81 |
| MVPC2 | Marlborough | MVPC | 12.76 | 6.12 |
| MVPC3 | Marlborough | MVPC | 7.93 | 35.42 |
| MZP1 | Marlborough | MZP | 13.46 | 0.11 |
| MZP2 | Marlborough | MZP | 13.46 | 0.98 |
| MZP3 | Marlborough | MZP | 13.48 | 0.86 |
| TARP1 | Martinborough | TARP | 13.2 | 0.02 |
| TARP2 | Martinborough | TARP | 13.26 | 3.68 |
| TARP3 | Martinborough | TARP | 13.21 | 2.5 |
| TETP1 | Martinborough | TETP | 13.14 | 0.1 |
| TETP2 | Martinborough | TETP | 13.36 | 0.06 |
| TETP3 | Martinborough | TETP | 13.54 | 0.45 |
| TMVP1 | Martinborough | TMVP | 9.63 | 28.69 |
| TMVP2 | Martinborough | TMVP | 11.73 | 10.76 |
| TMVP3 | Martinborough | TMVP | 11.67 | 16.19 |
| TPCP1 | Martinborough | TPCP | 11.04 | 16.86 |
| TPCP2 | Martinborough | TPCP | 8.66 | 32.67 |
| TPCP3 | Martinborough | TPCP | 13.03 | 6.1 |
| TPYP1 | Martinborough | TPYP | 12.74 | 3.6 |
| TPYP2 | Martinborough | TPYP | 8.99 | 28.54 |
| TPYP3 | Martinborough | TPYP | 10.89 | 17.68 |
| TTMP1 | Martinborough | TTMP | 8.86 | 33.47 |
| TTMP2 | Martinborough | TTMP | 12.07 | 12.14 |
| TTMP3 | Martinborough | TTMP | 8.72 | 34.49 |

**Methodology**

***Optical density curve development***

Given the number of samples being assessed (17), an Optical Density (OD) curve was developed to determine the inoculation concentration for all ferments. The curve was created using communities from TPYP and CQRP, TPYP comprised of *Hanseniaspora uvarum* + *Candida zemplinina* and CQRP *S. cerevisiae* exclusively. Three different yeast species were deliberately used to account for any potential variation in optical density due to variation in yeast shape.

The communities were revived using the methodology outlined previously. Seventy-five microlitres of aliquot was taken from each cell aseptically and transferred to a sterile Falcon tube. The tubes were then centrifuged at 3000 RCF in Eppendorf Centrifuge 5810R for 5 minutes to pellet the cells. The YPD liquid was removed, and the cells were resuspended with 5mL of sterile Milliq water.

Manual cell counts were performed using a hemocytometer to manually determine the CFU/mL. Serial dilutions were performed, and OD measurements were taken at 600 nm wavelength using VWR UV-1600PC Spectrophotometer. Standard curves were calculated to determine the relationship between the CFU/mL, as determined based on the hemocytometer cell counts and amount of dilution, and the Optical Density of the cultures at 600 nm (OD_600_). Linear trendlines were fitted to the data for each sample tested (Supplemental Figure 4, Supplemental Figure 5, Supplemental Figure 6). The equations were averaged together and used to create a chart correlating OD_600nm_ measurements to CFU/mL. This linear model was utilised to determine inoculation volumes for all fermentations (Supplemental Table 4).

**Supplemental Table 4:** Points along optical density curve

| OD_600_ Measurement | Estimated CFU/mL | Volume to Achieve 2.50x10^06^ CFU/mL of Inoculum (mL) |
| --- | --- | --- |
| 0.1 | 9.10x10^07^ | 5.496 |
| 0.11 | 1.01x10^08^ | 4.951 |
| 0.12 | 1.11x10^08^ | 4.505 |
| 0.13 | 1.21x10^08^ | 4.133 |
| 0.14 | 1.31x10^08^ | 3.817 |
| 0.15 | 1.41x10^08^ | 3.547 |
| 0.16 | 1.51x10^08^ | 3.312 |
| 0.17 | 1.61x10^08^ | 3.106 |
| 0.18 | 1.71x10^08^ | 2.924 |
| 0.19 | 1.81x10^08^ | 2.763 |
| 0.2 | 1.91x10^08^ | 2.618 |
| 0.21 | 2.01x10^08^ | 2.488 |
| 0.22 | 2.11x10^08^ | 2.370 |
| 0.23 | 2.21x10^08^ | 2.263 |
| 0.24 | 2.31x10^08^ | 2.165 |
| 0.25 | 2.41x10^08^ | 2.075 |
| 0.26 | 2.51x10^08^ | 1.992 |
| 0.27 | 2.61x10^08^ | 1.916 |
| 0.28 | 2.71x10^08^ | 1.845 |
| 0.29 | 2.81x10^08^ | 1.779 |
| 0.3 | 2.91x10^08^ | 1.718 |
| 0.31 | 3.01x10^08^ | 1.661 |
| 0.32 | 3.11x10^08^ | 1.608 |
| 0.33 | 3.21x10^08^ | 1.558 |
| 0.34 | 3.31x10^08^ | 1.511 |
| 0.35 | 3.41x10^08^ | 1.466 |
| 0.36 | 3.51x10^08^ | 1.425 |
| 0.37 | 3.61x10^08^ | 1.385 |
| 0.38 | 3.71x10^08^ | 1.348 |
| 0.39 | 3.81x10^08^ | 1.312 |
| 0.4 | 3.91x10^08^ | 1.279 |
| 0.41 | 4.01x10^08^ | 1.247 |
| 0.42 | 4.11x10^08^ | 1.217 |
| 0.43 | 4.21x10^08^ | 1.188 |
| 0.44 | 4.31x10^08^ | 1.160 |
| 0.45 | 4.41x10^08^ | 1.134 |
| 0.46 | 4.51x10^08^ | 1.109 |
| 0.47 | 4.61x10^08^ | 1.085 |
| 0.48 | 4.71x10^08^ | 1.062 |
| 0.49 | 4.81x10^08^ | 1.040 |
| 0.5 | 4.91x10^08^ | 1.018 |
